# Discovery of Euryhaline Phycoerythrobilin-Containing *Synechococcus* and Its Mechanisms for Adaptation to Estuarine Environments

**DOI:** 10.1128/mSystems.00842-20

**Published:** 2020-12-15

**Authors:** Xiaomin Xia, Puiyin Lee, Shunyan Cheung, Yanhong Lu, Hongbin Liu

**Affiliations:** a Key Laboratory of Tropical Marine Bio-resources and Ecology, South China Sea Institute of Oceanology, Chinese Academy of Sciences, Guangzhou, People’s Republic of China; b Southern Marine Science and Engineering Guangdong Laboratory (Guangzhou), Guangzhou, People’s Republic of China; c Department of Ocean Science, The Hong Kong University of Science and Technology, Hong Kong, China; d Hong Kong Branch of Southern Marine Science & Engineering Guangdong Laboratory, The Hong Kong University of Science and Technology, Hong Kong, China; United States Naval Research Laboratory

**Keywords:** euryhaline *Synechococcus*, channel protein, genome, transcriptome

## Abstract

*Synechococcus* are among the most abundant and widely distributed picocyanobacteria on earth. Cluster 5 phycoerythrobilin-containing (PEB-containing) *Synechococcus*, the major marine *Synechococcus*, were considered to prefer high salinity, and they are absent in estuarine ecosystems. However, we have detected PEB-containing *Synechococcus* in some low-salinity (<15-ppt) areas of the Pearl River estuary at an abundance up to 1.0 × 10^5^ cells ml^−1^. Two PEB-containing *Synechococcus* strains (HK01 and LTW-R) were isolated, and tests on them revealed their ability to cope with variations in the salinity (from 14 to 44 ppt). Phylogenetic analysis showed that HK01 belonged to a novel *Synechococcus* clade (HK1), whereas LTW-R was clustered with S5.2 strains. Whole-genome analysis revealed that a membrane channel protein with glycine zipper motifs is unique to euryhaline *Synechococcus*. The upregulation of this protein, the osmotic sensors, and the heat shock protein HSP20 and the downregulation of the osmolyte biosynthesis enable euryhaline *Synechococcus* to well adapt to the low and fluctuating salinity in the estuarine environment. In addition, decreasing the salinity in LTW-R strongly downregulated several important metabolic pathways, including photosynthesis, and the Calvin-Benson cycle, whereas its growth was not significantly affected. Moreover, obtaining PEB genes from horizontal gene transfer expands the light niche significantly for euryhaline *Synechococcus*. These results provided new insights into the life strategies and ecological function of marine PEB-containing *Synechococcus* under the unique environmental condition of estuarine waters, particularly in response to salinity variations.

**IMPORTANCE** Understanding the strategies developed by different microbial groups to adapt to specific niches is critical. Through genome and transcriptome analyses of two newly isolated novel euryhaline *Synechococcus* strains, this study revealed that cluster 5 phycoerythrobilin-containing *Synechococcus*, which are thought to be strictly marine strains, could be abundant in low-salinity waters of the Pearl River estuary (salinity <15 ppt) and explained the molecular mechanisms that enabled them to adapt the low and fluctuating salinity in the estuarine environment. This study expands current understanding on mechanisms involved in niche separation of marine *Synechococcus* lineages.

## Abstract (precis)

Understanding the strategies developed by different microbial groups to adapt to specific niches is critical. Through genome and transcriptome analyses of two newly isolated novel euryhaline *Synechococcus* strains, this study revealed that cluster 5 phycoerythrobilin-containing *Synechococcus*, which are thought to be strictly marine strains, could be abundant in low-salinity waters of the Pearl River estuary (salinity <15 ppt) and explained the molecular mechanisms that enabled them to adapt the low and fluctuating salinity in the estuarine environment.

## INTRODUCTION

Marine *Synechococcus* (cluster 5) are among the most abundant and widely distributed photosynthetic organisms in the global ocean (1–3). In addition to chlorophyll *a*, *Synechococcus* contain a set of phycobiliproteins as light-harvesting pigments. Based on the phycobilisome structure, three different types of *Synechococcus* have been identified. They are phycocyanobilin (PCB)-only, phycoerythrobilin (PEB)-only (containing PCB and PEB), and phycourobilin (PUB)-containing (containing PCB, PEB, and PUB) (4). Because both the latter two types contain PEB, they are also classed as being PEB-containing *Synechococcus*. On the other hand, using gene markers such as *petB* and *rpoC1*, cluster 5 *Synechococcus* have also been classified into three phylogenetic subclusters (S5.1, S5.2, and S5.3) and at least 20 clades (5, 6). The niche separation of *Synechococcus* phylogenetic lineages and pigment types in marine environments has been widely reported (5, 7–9). PCB-only *Synechococcus* are green in color, and these consist of euryhaline strains, which are affiliated with subcluster 5.2 (here called S5.2) and S5.1 clade VIII (4, 10). They are distributed in estuarine waters with high nutrient levels, high turbidity, and low salinity (4, 11, 12). In contrast, PEB-containing *Synechococcus* are red to pink in color, and these are mainly composed of S5.1 and S5.3 clades, which dominate coastal and oceanic waters (6, 9).

**FIG 1:**
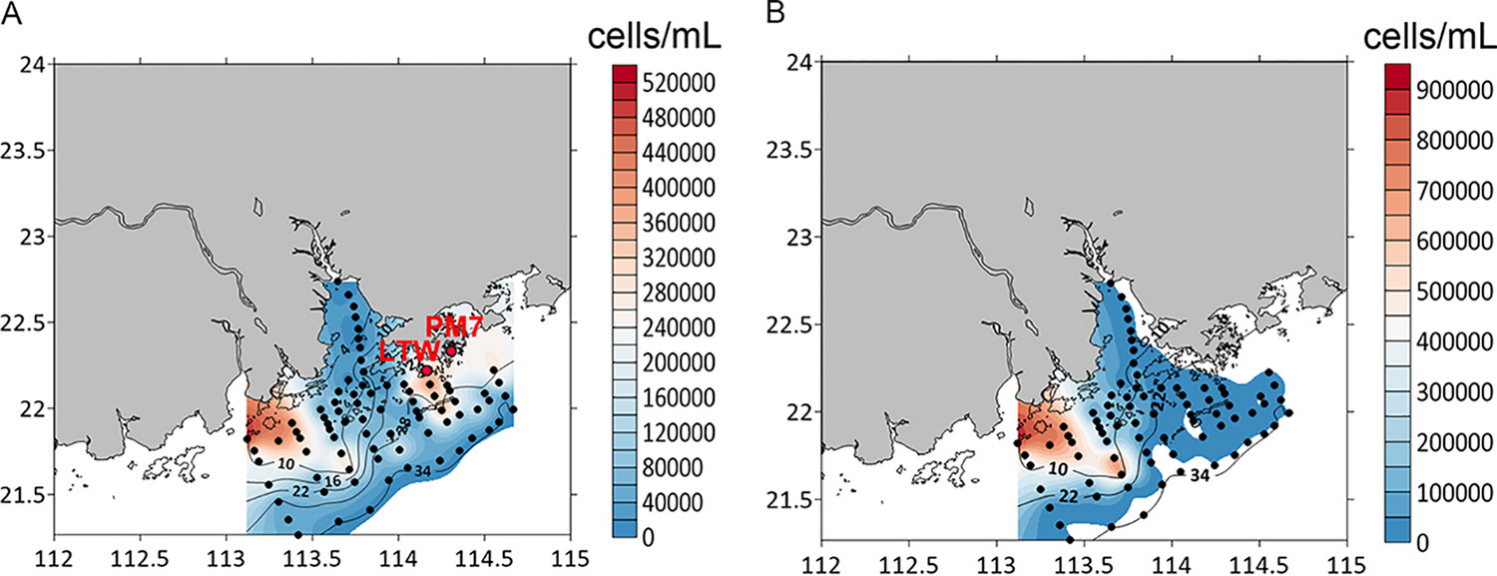
Distribution of PEB-containing (A) and PCB-only (B) *Synechococcus* in the Pearl River estuary in July 2014. The color bars indicate the different abundances of *Synechococcus* cells (evaluated using flow cytometry), and the contour lines indicate the different salinities of the surface water. Please note that the scale bars are different for the two plots. The maps were generated using the software Surfer V15.

Salinity is a barrier that separates freshwater and marine organisms (13–15). It has been suggested that marine-freshwater transitions in the microbial world are infrequent because most microbes cannot cope with variations in the environmental salinity (16). Bacteria are bounded by a porous cell wall and semipermeable cytoplasmic membrane, and so in an environment with high external osmotic pressure, they are likely to become dehydrated due to the efflux of water. In contrast, when the external osmotic pressure decreases, then there will be an influx of water across the cell wall and cytoplasmic membrane, which results in cell lysis. For this reason, bacteria have developed several mechanisms to deal with osmotic stress, including the salt-in-cytoplasm mechanism and organic-osmolyte mechanism (17). Some bacteria can regulate the concentration of their cytoplasmic solute by producing/excreting organic molecules (18), or they have transporters and protein channels to deal with variations in the salinity (19–23). Salinity has been reported to affect the growth and photosynthetic function of *Synechococcus* (10, 24). For example, it has long been believed that cluster 5 PEB-containing *Synechococcus* (including both the PEB-only and PUB-containing strains) are strictly marine strains, which cannot survive in estuaries due to the low salinity and high turbidity of the water (12). Indeed, in a previous study, we showed that different *Synechococcus* strains have various abilities to deal with salinity variations. We demonstrated that the growth of PCB-only strains was seldom affected by salinity changes whereas most PEB-containing strains were negatively influenced by a decrease in salinity, and in some cases these strains were unable to grow when the salinity was lower than 15 ppt (10). Similar results have also been reported for *Synechococcus* in the Baltic Sea (25). However, using a combination of sequencing and flow cytometry analysis, we recently observed that some cluster 5 PEB-containing *Synechococcus* might have developed the ability to cope with variations in the salinity and they can be found in estuarine waters (11). For example, in the Pearl River estuary during July 2014, the concentration of PEB-containing *Synechococcus* reached 1.0 × 10^5^ cells ml^−1^ at some of the sampling stations where the salinity was <15 ppt (Fig. 1). However, little is known about the mechanisms involved in the adaption to salinity of these euryhaline *Synechococcus*.

Whole-genome sequence analysis and transcriptomic analysis have provided new insights into the strategies developed by different *Synechococcus* phylogenetic lineages to adapt to specific niches. The first *Synechococcus* genome was sequenced in 2003 by Palenik et al. (26), and they found that clade III strain WH8102 adopted strategies such as a reduction of the regulatory machinery to save energy to help it adapt to oligotrophic waters. Researchers subsequently showed, by comparing the genome sequences of the coastal *Synechococcus* sp. strain CC9311 and the oligotrophic strain WH8102, that CC9311 has a greater capacity to sense and respond to changes in coastal environmental factors (e.g., high iron and copper concentrations) than WH8102 (27). Moreover, in *Synechococcus* sp. PCC7002 (cluster 3), the mechanisms involved in its acclimation to different growth conditions (such as temperature) have been revealed by transcriptomic analysis (28). Thus, a whole-genomic and transcriptomic comparison of strictly marine and euryhaline *Synechococcus* might help to reveal how the latter can adapt to stressful estuarine environments.

In this study, we isolated two novel euryhaline PEB-containing *Synechococcus* strains from Hong Kong riverine-influenced coastal waters. We sequenced their genomes using a combination of second- and third-generation sequencing technologies (i.e., Illumina HiSeq and PacBio, respectively) and made comparisons with published *Synechococcus* genomes in the National Center for Biotechnology Information (NCBI) database. Transcriptomic analysis was then applied to compare the gene expression levels under different salinities. In this way, we were able to identify the potential mechanisms used by euryhaline PEB-containing *Synechococcus* to adapt to estuarine environments.

## RESULTS AND DISCUSSION

### Pigment and salinity tolerance of *Synechococcus* sp. HK01 and LTW-R.

**FIG 2:**
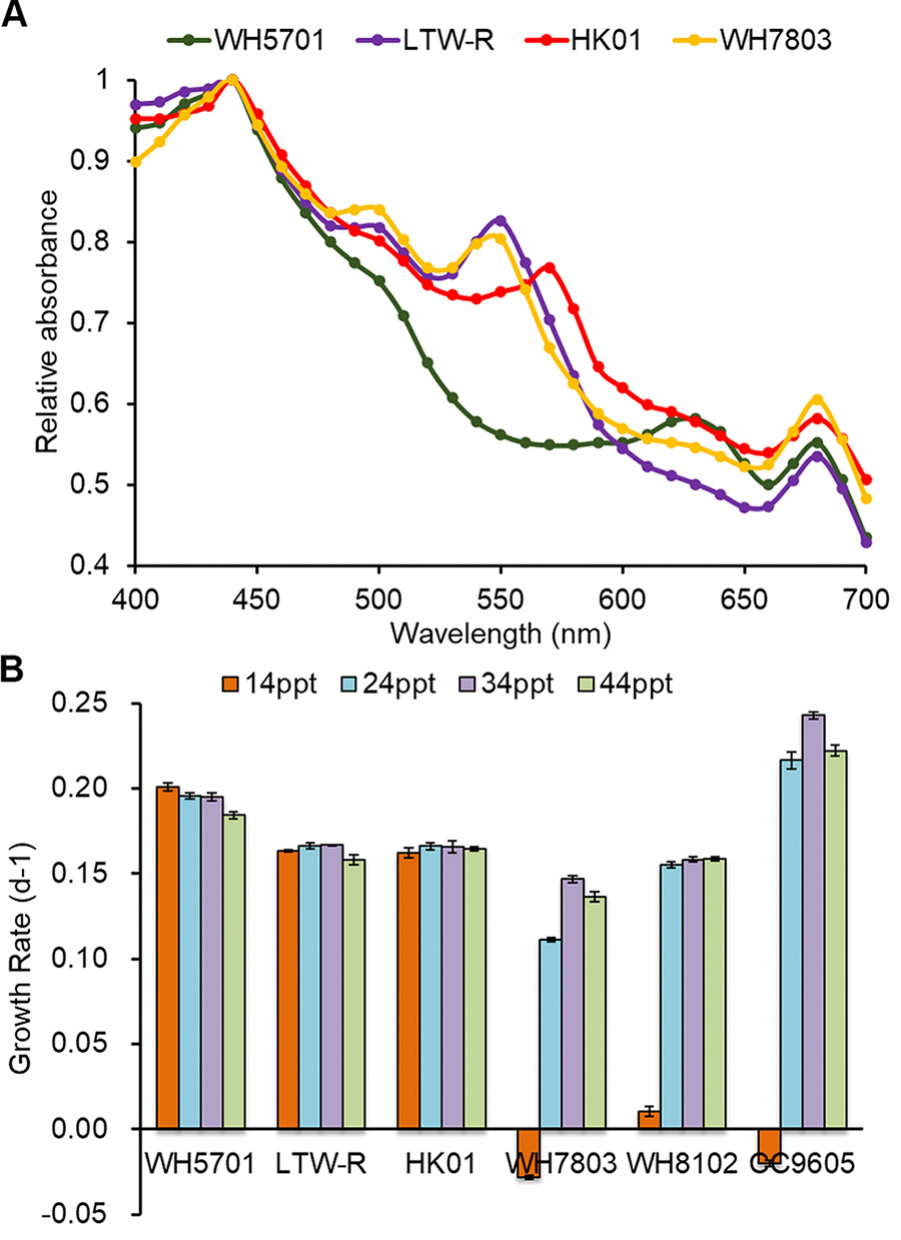
*In vivo* absorption spectra of HK01 and LTW-R (A) and their growth rates under different salinities (B). WH5701 and WH7803 were used as reference strains for the *in vivo* absorption spectrum measurement; WH5701 is a PCB-only *Synechococcus* whereas WH7803 is a PUB-containing *Synechococcus*.

HK01 is a PEB-only strain whereas LTW-R is a low-PUB-containing strain (Fig. 2A). HK01 and LTW-R both exhibited absorbance peaks at ∼440 nm and ∼670 nm (Fig. 2A), which indicates that these two strains contain PEB. However, the middle absorbance peak of HK01 occurred at 570 nm, and so it was red-shifted relative to that of LTW-R, which was at 550 nm (Fig. 2A). This is consistent with previous observations that PEB-only *Synechococcus* are well adapted to harvest light in fairly turbid waters where the photosynthetically active radiation (PAR) spectrum is likely shifted toward yellow/yellow-green light due to organic matter in suspension (5, 29, 30). Compared with HK01, LTW-R had an additional absorbance peak at 495 nm, indicating it contains PUB. This means that LTW-R can harvest light from wider PAR spectra than PEB-only strains, extending from blue-green to yellow-green (4, 5).

**FIG 3:**
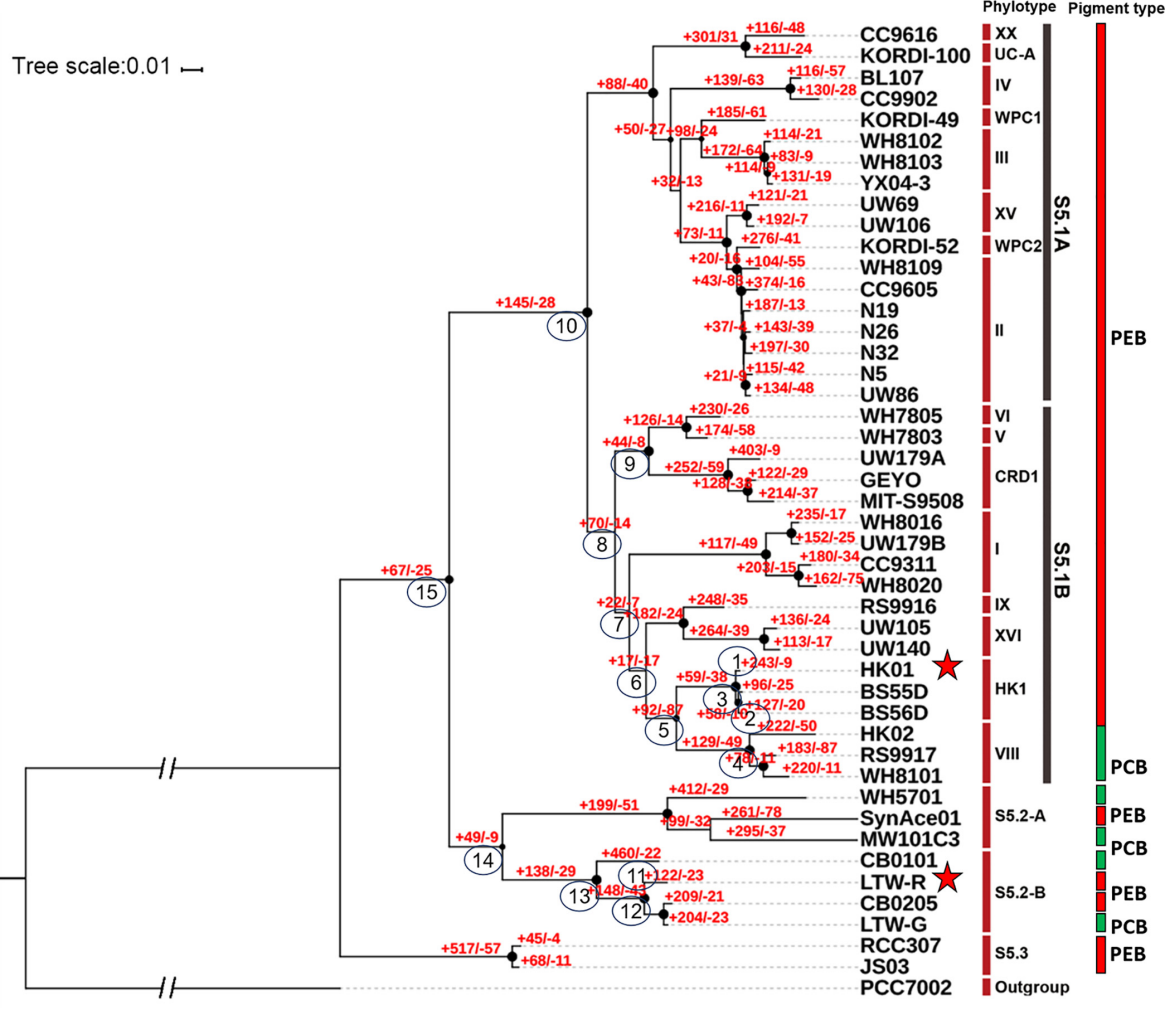
Phylogenetic analysis of HK01 and LTW-R based on 43 concatenated phylogenetically informative marker genes using the maximum likelihood method with a JTT model. HK01 and LTW-R are labeled with red stars. Orthologous group (OG) gain and loss numbers (in red) were evaluated using the Count software package and are labeled accordingly on the tree nodes and tips. The numbers in circles indicate the order of the nodes and tips, and the black dots in each node indicate bootstrap values of >70. Important genes shown to be involved in OG gain or loss events in HK01 and LTW-R are indicated in Fig. S1.

To examine the salt adaptation of HK01 and LTW-R, these together with WH5701 (S5.2, a euryhaline strain), WH7803 (clade V, a strictly marine strain), WH8102 (clade III, a strictly marine strain), and CC9605 (clade II, a strictly marine strain) were cultured in modified f/2 medium (without Na_2_SiO_4_) with the salinity ranging from 14 ppt to 44 ppt. Our results clearly showed that the typical strictly marine strains (WH7803, WH8102, and CC9605) had a relatively narrow spectrum of salinity tolerance (Fig. 2B), such that a salinity of 14 ppt resulted in a dramatic decline in the growth rate. This finding is consistent with previous studies, which reported that S5.1 strictly marine *Synechococcus* strains do not grow well in low-salinity environments (10, 31). We also showed that although HK01 and LTW-R are PEB-containing strains, they still coped well with low-salinity stress similarly to the typical euryhaline PCB-only strains, WH5701 (Fig. 2B) and CB0101 (see Fig. 2 and 3 in the work of Wang [31]). This indicates that HK01 and LTW-R are euryhaline PEB-containing *Synechococcus* strains. It should be noted that compared with PCB-only strains, both PEB-only and low-PUB-containing strains prefer less turbid water, resulting in a narrower distribution of the euryhaline PEB-containing *Synechococcus* in estuarine environments compared with PCB-only *Synechococcus*. Nevertheless, the discovery of euryhaline PEB-containing *Synechococcus* fills the gap between strictly marine PEB-containing and euryhaline PCB-only *Synechococcus*, in both evolutionary and biogeography perspectives.

### Phylogeny of HK01 and LTW-R.

Phylogenetic analysis of the *Synechococcu*s strains was based on 43 concatenated phylogenetically informative marker genes, including ribosomal proteins and RNA polymerase domains (Fig. 3) (32). The results showed that the 45 strains used in this study covered all three reported subclusters of marine *Synechococcus* (6, 33). HK01 together with BS55D and BS56D formed a new *Synechococcus* clade (HK1). BS55D and BS56D are both PEB-containing *Synechococcus* sp. strains, and they were isolated from a depth of 750 m. They are known to be able to survive in harsh mesopelagic environments (34). In addition, although clade VIII is the closest phylogenetic neighbor of clade HK1, they have different pigments, such that clade HK1 is a group of PEB-only *Synechococcus* whereas clade VIII is a group of euryhaline PCB-only *Synechococcus* (5). On the other hand, LTW-R was clustered with S5.2 strains CB0205 and CB0201 in the phylogenetic tree. We named this clade S5.2-B, a sister clade to S5.2-A, which comprises WH5701 (a PCB-only strain), Synace01 (a PEB-containing strain), and MW101C3 (a PCB-only strain) (Fig. 3). It is interesting that clades S5.2-A and S5.2-B include both PEB-containing and PCB-only strains (Fig. 3). This new finding suggests that S5.2 developed different pigment types to adapt to various light niches, although they are confined in estuaries (5, 10).

10.1128/mSystems.00842-20.1 FIG S1 Important genes involved in the gain (in yellow) or loss (blue) events in the LTW-R clade (A), S5.1 euryhaline clade (B), and HK01 clade (C). The order of nodes corresponds to the circled numbers in Fig. 3, and the function of the genes can be found in Data Set S1. Asterisks indicate transporters/antiporters. The letter following each gene name shows the functional category according to the COG database. C, Energy production and conversion; D, Cell cycle control, cell division, chromosome partitioning; E, Amino acid transport and metabolism; F, Nucleotide transport and metabolism; G, Carbohydrate transport and metabolism; H, Coenzyme transport and metabolism; J, Translation, ribosomal structure and biogenesis; M, Cell wall/membrane/envelope biogenesis; O, Posttranslational modification, protein turnover, chaperones; P, Inorganic ion transport and metabolism; Q, Secondary metabolites biosynthesis, transport and catabolism; T, Signal transduction mechanisms; U, Intracellular trafficking, secretion, and vesicular transport; V, Defense mechanisms. Download FIG S1, TIF file, 2.9 MB. Copyright © 2020 Xia et al. 2020 Xia et al. This content is distributed under the terms of the Creative Commons Attribution 4.0 International license.

Compared with the ancestor (node 15, Fig. 3), LTW-R and HK01 both obtained some important traits via horizontal gene transfer (HGT), such as urea utilization, sugar transport, chloride transport, and copper homeostasis and tolerance, which help them to adapt well to an estuarine environment (see Fig. S1 in the supplemental material). LTW-R also gained the *sdmt* and *gsmt* genes, which are involved in the biosynthesis of the osmotic component betaine (35, 36). This suggests that LTW-R might use different strategies for dealing with high osmotic pressures than the other S5.2 strains (Fig. S1A).

### Properties of the HK01 and LTW-R genomes.

The genomes of HK01 and LTW-R were assembled using a combination of PacBio long-read sequencing and Illumina short-read sequencing. They each contain a single contig, which is 2.48 Mb and 2.42 Mb, respectively, in size (Table 1). LTW-R has 52 RNA genes, which is more than HK01 and strictly marine strains. The GC content of HK01 and LTW-R is 61.2% and 62.6%, respectively; these values are higher than strictly marine PEB-containing *Synechococcus* but lower than PCB-only strains (Fig. S2). These results support the suggestion that a low genomic GC content might be an adaptation to nitrogen limitation (37). This is because strictly marine PEB-containing strains mainly dominate in oceanic waters where the nitrogen concentration is often lower than in estuarine waters. In addition, it has been suggested that streamlining selection can drive genome reduction in low-nutrient environments as the small genome size and fewer gene duplications might provide an adaptive advantage to life in the oligotrophic ocean (38, 39). However, some oceanic *Synechococcus* strains, such as KORDI-100 and Synace01, which are distributed in low-nutrient environments, did not display a smaller genome size than the coastal or estuarine strains, so the size distribution pattern of *Synechococcus* genomes does not support this theory (Fig. S2).

**TABLE 1:** Comparison of genome characteristics of typical euryhaline and strictly marine *Synechococcus*^*a*^

	Euryhaline strains				Strictly marine strains		
	*LTW-R*	WH5701	CB0101	*HK01*	WH7803	WH8102	CC9605
Lineage	S5.2	S5.2	S5.2	HK1	V	III	II
Contigs	1 (circular)	111	1	1	1	1	1
Genome size (Mb)	2.42	3.03	2.79	2.48	2.37	2.43	2.51
No. of coding sequences	2,556	3,323	3,131	2,744	2,637	2,698	2,947
No. of RNAs	52	56	52	50	46	50	50
Accession no.	CP059060	NZ_CH724159	CP039373	CP059059	NC009481	BX548020	CP000110
GC content (%)	62.6	65.5	64.1	61.2	60.2	59.4	59.2

a LTW-R and HK01 are labeled in italics for emphasis.

10.1128/mSystems.00842-20.2 FIG S2 GC content and size of the *Synechococcus* genomes. The green circles indicate the PCB-only *Synechococcus*, whereas the red circles are the PEB-containing *Synechococcus*. HK01 and LTW-R are shown by red triangles. Download FIG S2, TIF file, 2.5 MB. Copyright © 2020 Xia et al. 2020 Xia et al. This content is distributed under the terms of the Creative Commons Attribution 4.0 International license.

Gene prediction of the HK01 and LTW-R genomes resulted in 2,744 and 2,556 coding sequences (CDS), respectively. In total, 1,707 and 1,628 CDS of HK01 and LTW-R were respectively assigned into 25 Cluster of Orthologous Groups (COG) function catalogs (Fig. S3). Genes involved in translation, ribosomal structure, and biogenesis; cell wall/membrane/envelope biogenesis; energy production and conversion; and coenzyme transport and metabolism were more abundant than other COG functions (Fig. S3). In addition, although LTW-R is affiliated with S5.2, it has fewer coding sequences than the other two S5.2 strains, WH5701 and CB0101, but is similar to the S5.1 *Synechococcus* strains (Table 1). In general, apart from LTW-R, euryhaline strains have more CDS involved in transcription, replication, recombination, and repair than the strictly marine strains (unpaired *t* test, *P* < 0.05) (Fig. S4).

10.1128/mSystems.00842-20.3 FIG S3 Circular genome map of HK01 and LTW-R (A) and the number of ORFs in each COG category (B). The GC content, GC skew, and predicted open reading frame (ORF) are shown. Download FIG S3, TIF file, 0.6 MB. Copyright © 2020 Xia et al. 2020 Xia et al. This content is distributed under the terms of the Creative Commons Attribution 4.0 International license.

10.1128/mSystems.00842-20.4 FIG S4 Functional classification of the coding sequences of the various *Synechococcus* strains. Coding sequences that were assigned into several different COG categories were not “dereplicated” but left in the categories assigned. Download FIG S4, TIF file, 2.7 MB. Copyright © 2020 Xia et al. 2020 Xia et al. This content is distributed under the terms of the Creative Commons Attribution 4.0 International license.

**FIG 4:**
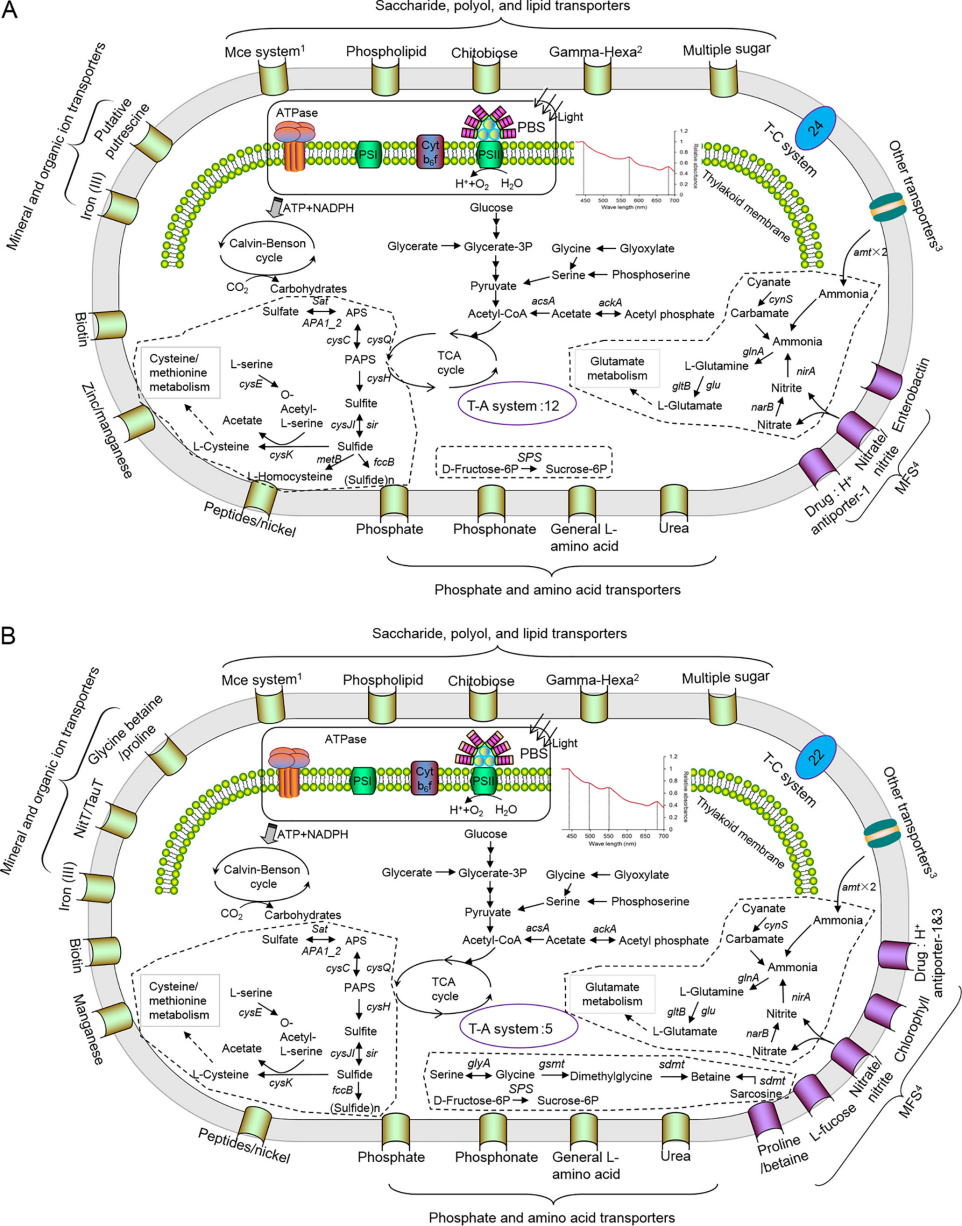
Metabolic pathways in HK01 (A) and LTW-R (B) based on annotation of eggNOG-Mapper and BlastKOALA. In this figure, only ABC transporters that contain substrate-binding protein, permease protein, and ATP-binding protein are shown. T-A system, toxin and antitoxin system; T-C system, two-component system; TCA, tricarboxylic acid. ^1^Mce system, phospholipid/cholesterol/gamma-hexachlorocyclohexane (HCH) transporter. ^2^Gamma-Hexa, gamma-hexachlorocyclhexane transporter. ^3^Other transporters; for details, see Data Set S1. ^4^MFS, major facilitator superfamily.

The HK01 and LTW-R genomes had complete ABC transporters involved in the import of organic materials, such as amino acids and saccharides, which suggests that they might grow as a mixotroph in marine environments (Fig. 4) (40, 41). This might explain why a considerable number of *Cyanobacteria* have been observed in some dark oceans (34). However, we found that the euryhaline strains and oceanic strains had different preferences for amino acid utilization. For example, the euryhaline strains HK01 and LTW-R had an ABC transporter for general l-amino acid transportation, whereas a typical S5.2 strain such as CB0101 had a transporter for branched-chain amino acids, and WH8102 had transporters for both types of amino acids (Fig. 4 and Fig. S5).

10.1128/mSystems.00842-20.5 FIG S5 Metabolic pathways in WH8102 (A) and CB0101 (B). T-A system, toxin and antitoxin system. T-C system, two component system. Only ABC transporters with substrate-binding protein, permease protein, and ATP-binding protein are shown. ^1^Mce system, phospholipid/cholesterol/gamma-HCH transporter. ^2^Gamma-Hexa, gamma-hexachlorocyclohexane transporter. ^3^Other transporters, for details see Data Set S1. ^4^MFS, major facilitator superfamily. Download FIG S5, TIF file, 2.8 MB. Copyright © 2020 Xia et al. 2020 Xia et al. This content is distributed under the terms of the Creative Commons Attribution 4.0 International license.

All euryhaline strains, including LTW-R and HK01, have an NNP (nitrate-nitrite porter), which belongs to the major facilitator superfamily (MFS) (42) for the transportation of inorganic nitrogen (Fig. 4 and Fig. S5). This is different from the typical oceanic strain WH8102, which has both an ABC-type nitrate transporter (NRT) and NNP. Ohashi et al. reported that NRT genes are absent in the WH8102 genome (43). However, NRT genes (*nrtA*, *nrtB*, and *nrtC*) were all detected in the WH8102 genome in the present study. It has been suggested that the ABC-type NRT is mainly distributed in freshwater strains of cyanobacteria, and it transports both nitrate and nitrite with high affinity (44). In contrast, the MFS-type NNP has much lower affinity for nitrite than for nitrate (45), and this is widely present in marine *Synechococcus* (46, 47), suggesting that marine *Synechococcus* prefer nitrate to nitrite. Ammonia is often highly abundant in estuarine waters, and it has a negative effect on the activity of the ABC-type NRT (43). This might lead to a loss of ABC-type NRT genes in euryhaline strains. However, compared with oceanic *Synechococcus* strains, the euryhaline strains have one more copy of the *amtB* gene, which is involved in ammonia transportation (Fig. 4 and Fig. S5). This might help them to utilize ammonia more efficiently in the coastal waters. Hence, our results suggest euryhaline *Synechococcus* and strictly marine *Synechococcus* have evolved distinct mechanisms to utilize different inorganic nitrogen sources.

### Pigment operon structure of HK01 and LTW-R.

**FIG 5:**
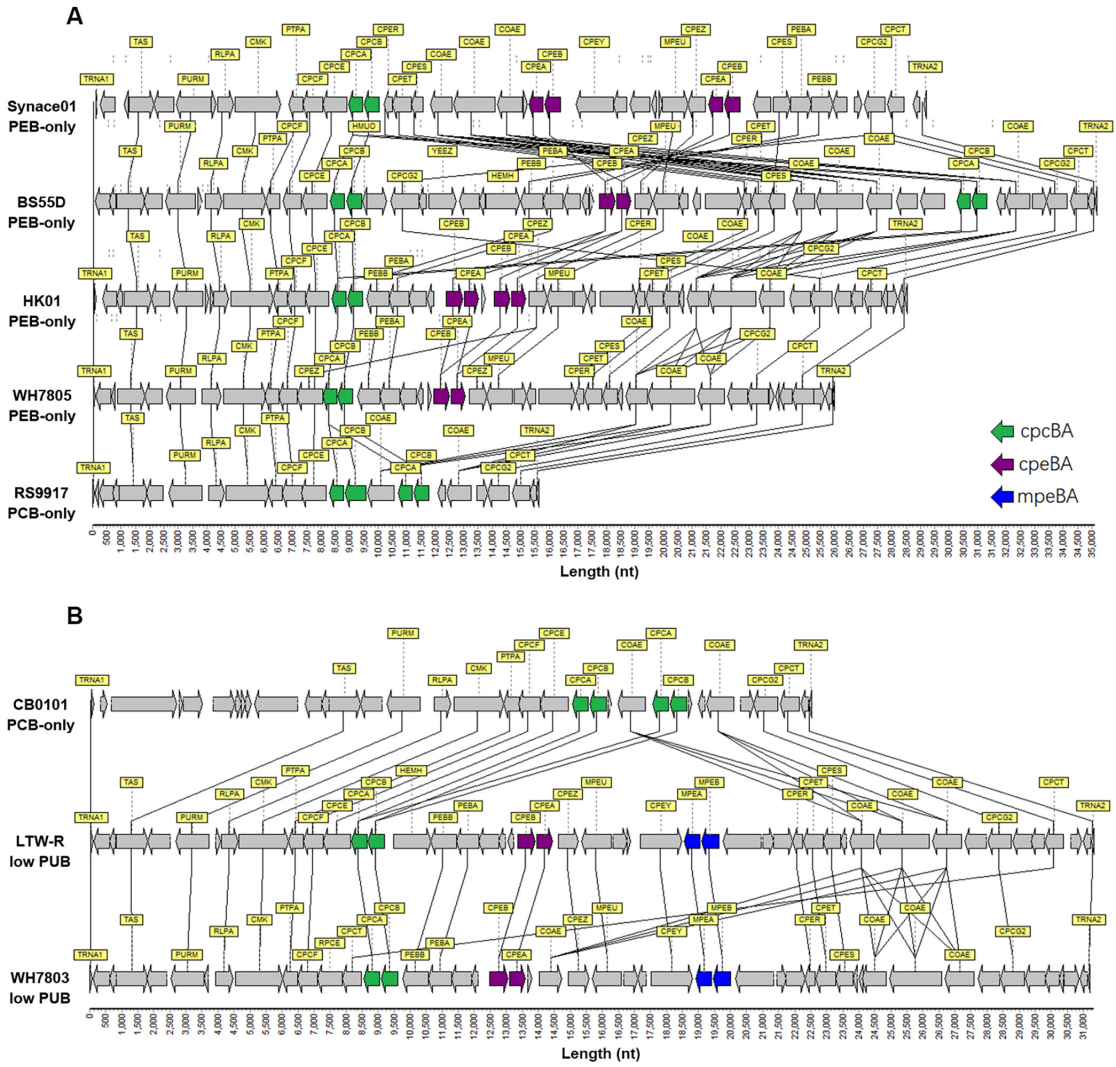
Comparison of the pigment operon structures of HK01 (A) and LTW-R (B) with those of other *Synechococcus* strains. The pigment operon structure of HK01 was compared with three other PEB-only strains (Synace01, BS55D, and WH7805), as well as the PCB-only strain (RS9917), which is phylogenetically related to HK01. The pigment operon structure of LTW-R was compared with those of a PCB-only strain (CB0101) and a low-PUB strain (WH7803). LTW-R and CB0101 are phylogenetically affiliated with S5.2-B. Synace01 is an S5.2-A PEB-only strain; BS55D is an S5.1-clade HK1 PEB-only strain; WH7805 is an S5.1-clade VI PEB-only strain; RS9917 is an S5.1-clade VIII PCB-only strain; CB0101 is an S5.2-B PCB-only strain; WH7803 is an S5.1-clade V low-PUB-containing strain.

The pigment operon structure of HK01 (PEB-only) was compared with that of PCB-only and other PEB-only strains (Fig. 5A). It is interesting that although HK01 is affiliated with the same clade as BS55D and they are PEB-only strains, HK01 has one more copy of the *cpeBA* gene (coding for phycoerythrin) and one fewer copy of the *cpcBA* gene (coding for phycocyanin) than BS55D (Fig. 5A). Besides HK01 and BS55D, Synace01 is also a PEB-only strain (48). It has two copies of the *cpeBA* gene like HK01, while the order and direction of *cpeBA* in the pigment operon of Synace01 are different from those of HK01 (Fig. 5A). WH7805, another PEB-only strain, has only one copy of each of the *cpeBA* and *cpcBA* genes. PEB-only strains are known to have different copy numbers of the *cpeBA* and *cpcBA* genes, which might result in different pigment protein structures and hence different light absorbance properties (e.g., PCB/PEB).

The pigment operon of LTW-R was also studied (Fig. 5B). LTW-R has one copy of each of the *cpcBA*, *cpeBA*, and *mpeBA* (coding for phycourobilin) genes. This supports the *in vivo* absorption spectrum analysis results, which indicate that LTW-R is a PUB-containing *Synechococcus*. The pigment operon structure of LTW-R is different from most other S5.2 strains but similar to the low-PUB-containing strains (Fig. 5B). An HGT analysis of *cpeB* in various *Synechococcus* strains showed that this gene was highly similar in LTW-R and in the S5.1 low-PUB-containing strain WH7803, indicating HGT of pigment genes among the *Synechococcus* lineages (Fig. S1 and S6). This finding was also supported by the analysis of the pigment operon structure of LTW-R and WH7803 (Fig. 5B). HGT analysis also suggested that HK1 clade strains might gain their *cpeB* gene from the close relatives of the S5.2 strain Synace01 (Fig. S6). These results suggest that HGT of pigment genes occurs widely among *Synechococcus* lineages, and euryhaline PEB-containing *Synechococcus* probably developed from euryhaline PCB-only *Synechococcus* which gained PEB genes via horizontal gene transfer (5, 49).

10.1128/mSystems.00842-20.6 FIG S6 Horizontal gene transfer (HGT) analysis of the *Synechococcus* pigment gene *cpeB.* HGT events were predicted using HGT-Detection. The red lines indicate possible horizontal transfer of the *cpeB* gene between *Synechococcus* strains. Numbers on HGTs indicate their order of inference. HGT bootstrap scores are indicated near the numbers of the corresponding HGTs. Download FIG S6, TIF file, 2.7 MB. Copyright © 2020 Xia et al. 2020 Xia et al. This content is distributed under the terms of the Creative Commons Attribution 4.0 International license.

### Salinity adaptation strategy of HK01 and LTW-R.

HK01 and LTW-R both displayed a high level of ability to deal with variations in salinity (Fig. 2B). It has been suggested that *Synechococcus* strains can adapt to a high salinity via a basic mechanism, which involves preventing inorganic salts from entering the cell and utilizing organic osmolytes to balance the high salinity of the environment (25, 48). The organic osmolytes commonly used by *Synechococcus* strains are sucrose, trehalose, glucosylglycerol, and glycine betaine (25). Our genomic analysis of euryhaline and strictly marine strains showed that the *SPS*, *ggpS*, and *STPA* genes, which are involved in synthesizing sucrose, glucosylglycerol, and trehalose, were present in all the genomes investigated, whereas the betaine-synthesizing genes *sdmt* and *gsmt* were distributed mainly in the strictly marine strains (Table 2). These results suggest that euryhaline strains and oceanic strains utilize different osmolytes for salinity acclimation (25). However, it is interesting that although LTW-R is a euryhaline strain, it also contains a copy of each of the *sdmt* and *gsmt* genes (Table 2) (50). The transcripts of these two genes were increased 2.60 and 2.87 times, respectively, at high salinity, which suggests that they might play a significant role in the salinity adaptation of LTW-R. It has also been reported that *mrp* (multiple resistance and pH adaptation) gene clusters are involved in salt stress tolerance of *Synechococcus* cultures (48). However, no *mrp* gene clusters were found in the LTW-R genome (euryhaline), although they were present in the WH7803 genome (strictly marine) (Data Set S1). This indicates that the *mrp* gene cluster might not be related to salinity adaptation.

**TABLE 2:** *Synechococcus* genes involved in the synthesis of compatible solutes used in salt acclimation^*a*^

	Euryhaline strains				Strictly marine strains		
	*LTW-R*	WH5701	CB0101	*HK01*	WH7803	WH8102	CC9605
*SPS* (sucrose)	1	1	1	1	1	1	1
*ggpS* (glucosylglycerol)	2	3	2	1	1	1	1
*STPA* (glucosylglycerol)	1	1	1	1	1	1	1
*sdmt* (betaine)	1	0	0	0	1	1	1
*gsmt* (betaine)	1	0	0	0	1	1	1

a The euryhaline strains LTW-R and HK01 are labeled in italics for emphasis.

10.1128/mSystems.00842-20.10 DATA SET S1 Excel file containing sequencing information, annotation of HK01 and LTW-R, function annotation of genes listed in Fig. S1, genes of LTW-R which were significantly changed in the low-salinity treatments, and transporter genes of euryhaline and oceanic *Synechococcus* strains. Download Data Set S1, XLSX file, 0.9 MB. Copyright © 2020 Xia et al. 2020 Xia et al. This content is distributed under the terms of the Creative Commons Attribution 4.0 International license.

**FIG 6:**
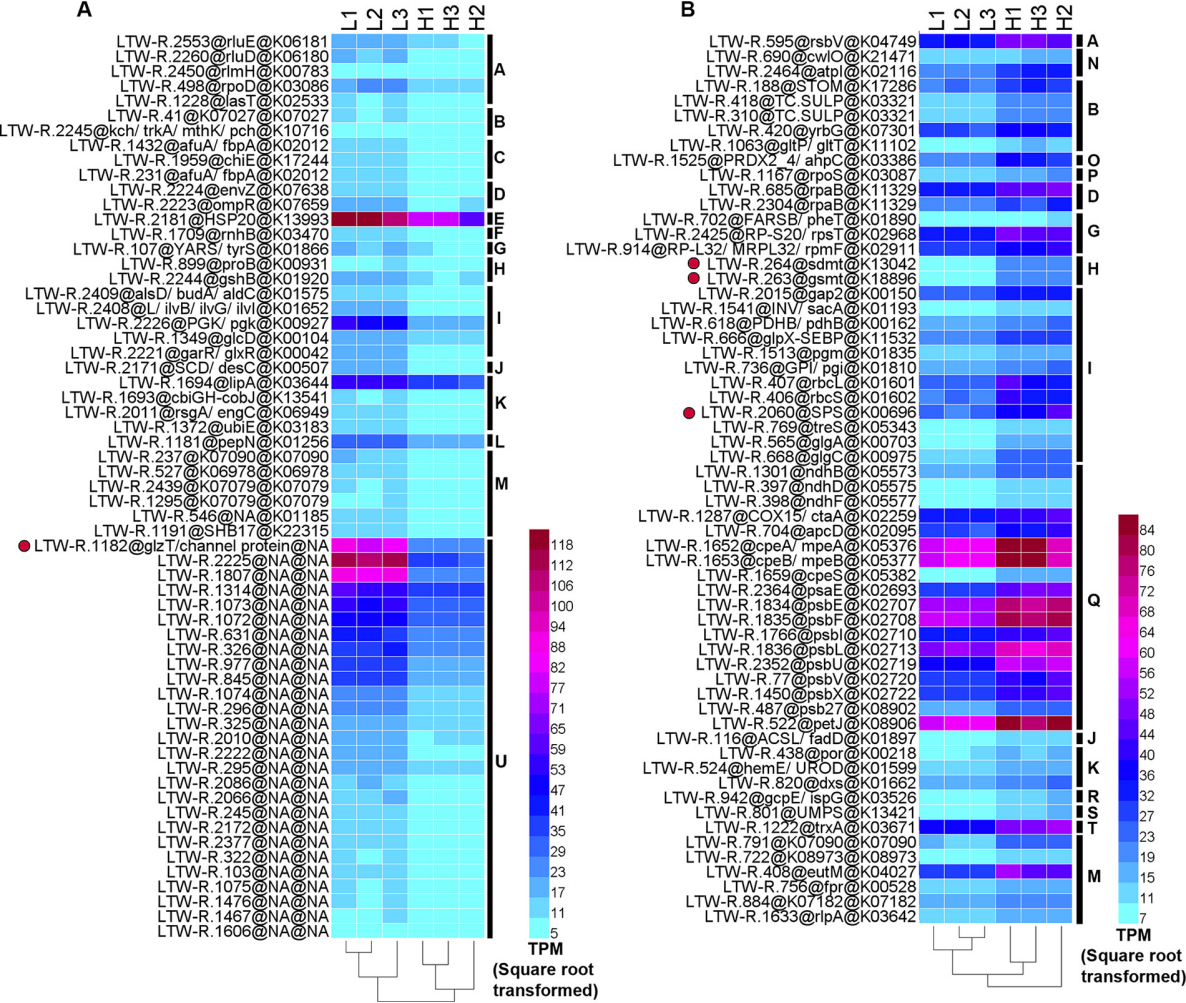
Heatmaps showing LTW-R genes that were significantly upregulated (A) or downregulated (B) in the low-salinity treatments. Low-salinity treatments (10 ppt), L1, L2, and L3; high-salinity controls (33 ppt), H1, H2, and H3. Genes with >2-fold change were considered to be strongly affected by the decrease in salinity. The color bars indicate the abundance (TPM, transcripts per million) of each gene. The data were square root transformed. Strongly downregulated unclassified genes and genes with an abundance lower than 100 TPM in all samples were not shown. Osmolyte biosynthesis genes and important channel genes are labeled with red circles. The black bars on the right indicate the different KEGG functions as follows: A, Protein families: genetic information processing; B, Protein families: signaling and cellular processes; C, Membrane transport; D, Signal transduction; E, Folding, sorting, and degradation; F, Replication and repair; G, Translation; H, Amino acid metabolism; I, Carbohydrate metabolism; J, Lipid metabolism; K, Metabolism of cofactors and vitamins; L, Metabolism of other amino acids; M, Poorly characterized; N, Protein families: metabolism; O, Cell growth and death; P, Cellular community—prokaryotes; Q, Energy metabolism; R, Metabolism of terpenoids and polyketides; S, Nucleotide metabolism; T, Chaperones and folding catalysts; U, unknown KEGG functions.

**FIG 7:**
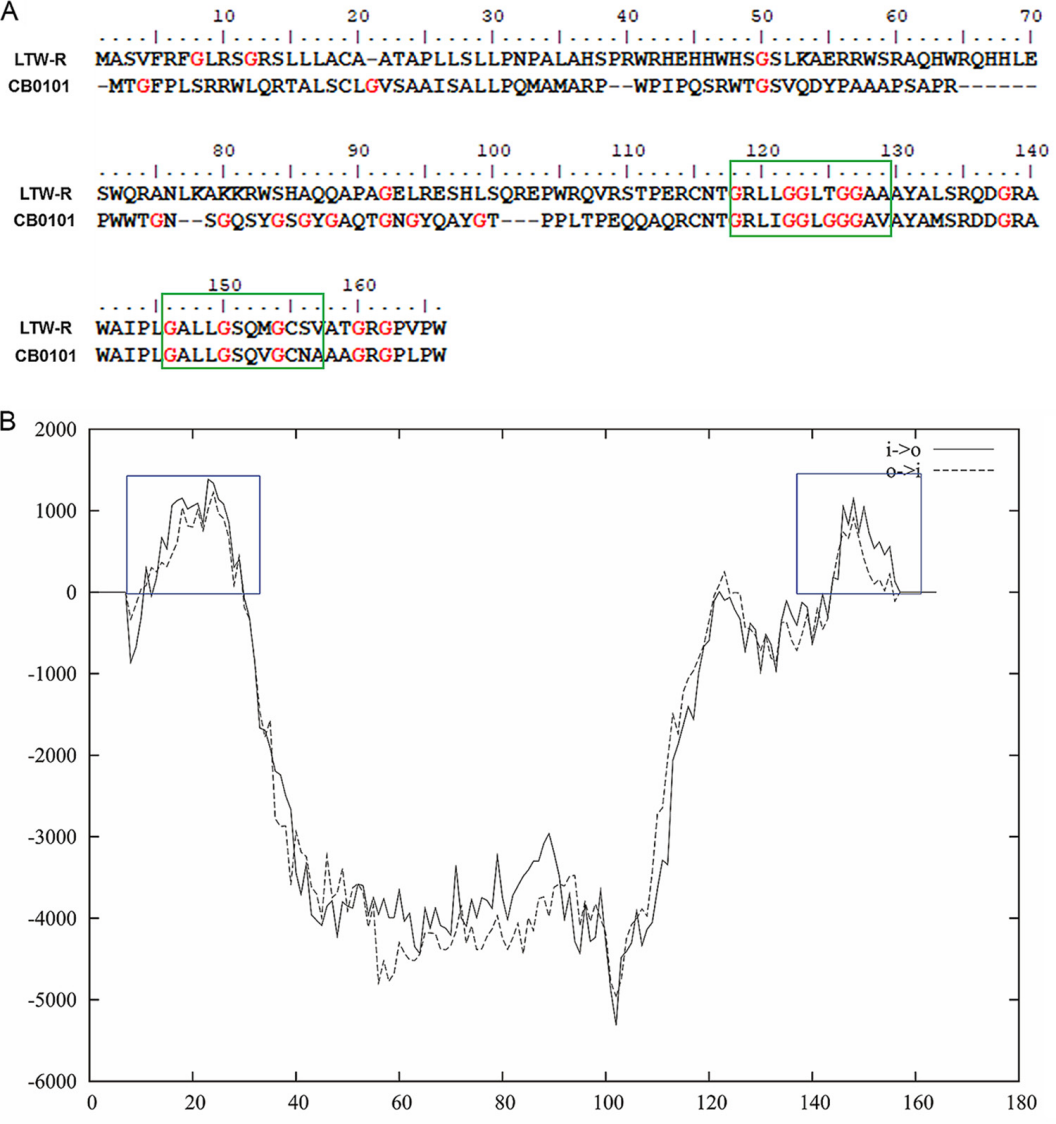
Typical glycine zippers (A) and transmembrane helices (B) predicted in ORF LTW-R.1182. This ORF is the representative sequence of OG cluster 2196 (named the *glzT* gene in this study), which is found only in euryhaline strains (see Table S1). The transcript of this ORF was upregulated 9.06-fold under the low-salinity condition. The green rectangles in panel A show typical glycine zipper sequences, and the blue squares in panel B show the helices predicted by TMpred.

To identify genes that might be involved in low-salinity adaption, we compared the genomes of HK01 and LTW-R with that of a typical euryhaline *Synechococcus* strain (i.e., CB0101) and with three strictly marine strains (i.e., CC9605, WH7803, and WH8102). Thirty orthologous groups (OGs) were unique to all of the euryhaline strains (Table S1 and Fig. S7). These gene clusters were involved in processes such as the biosynthesis of urease accessory protein, cobalt-zinc-cadmium efflux system protein, and the inorganic phosphate transporter. Four of these 30 OGs were significantly and strongly upregulated under low-salinity conditions (Table 3). The transcript of OG cluster 2196 (which was named the *glzT* gene in this study and is represented by open reading frame [ORF] LTW-R.1182) increased more than 9-fold when the salinity decreased, and thus, it was one of the most abundant transcripts under the low-salinity condition (Table 3 and Fig. 6A). However, both the GO and KEGG annotations indicated that the gene had an unknown function (Table 3). Analysis of its amino acid sequence showed that it contained two typical glycine zipper motifs (GXXXGXXXG) (Fig. 7) which are indicators of channel proteins (51). TMpred also predicted that there are two transmembrane helices in the ORF LTW-R.1182 (Fig. 7B). These results suggest that the *glzT* gene might encode a transmembrane protein that forms a channel, which plays a significant role in the low-salinity adaptation of *Synechococcus*. In addition, the osmotic sensor genes, *envZ* and *ormF*, were detected in the LTW-R genome, and the expression of both was also upregulated under the low-salinity condition (Fig. 6A). Thus, we suggest that to adapt to low-salinity environments, euryhaline strains first sense an upshift in the osmotic pressure of the cytoplasm via the use of osmotic sensor proteins, and then they activate their glycine zipper channel protein to pump the osmotic components or ions out of the cell to maintain the osmotic balance, and at the same time they decrease the biosynthesis of osmolytes and import of sugar (Fig. 6B).

**TABLE 3:** OGs found in all the euryhaline strains and transcripts alone, which were significantly changed in the low-salinity treatments

OG_name	Go_annotation	Ko no.	Gene_name	Function	LTW-R ORF	Fold change^*a*^	Mean abundance (TPM)^*b*^
cluster2196	N/A^*d*^	N/A	N/A	Glycine zipper 2TM domain-containing protein^*c*^	LTW-R.1182	9.06	3634.02
cluster2202	N/A	K08680	N/A	N/A	LTW-R.388	2.18	58.53
cluster2213	GO:0001522; P:pseudouridine synthesis; IEA:InterPro	K06181	*rluE*	23S rRNA pseudouridine2457 synthase	LTW-R.2553	2.03	217.60
cluster2231	N/A	K07090	K07090	Uncharacterized protein	LTW-R.237	3.37	167.28

a Fold change of the gene transcript when LTW-R cells were cultured at low salinity.

b Mean transcript abundance (TPM) of genes in LTW-R cells calculated from the 6 transcriptome samples.

c Function predicted by this study.

d N/A, not available.

10.1128/mSystems.00842-20.7 FIG S7 Distribution of the clusters of likely ortholog genes (CLOGs) across the euryhaline and strictly marine strains. Left panel shows the presence (purple) or absence (gray) of CLOGs. Right panel shows the protein count of each CLOG. The red star indicates CLOGs that are found only in the euryhaline strains. The annotation of these CLOGs is shown in Table S1. Download FIG S7, TIF file, 2.5 MB. Copyright © 2020 Xia et al. 2020 Xia et al. This content is distributed under the terms of the Creative Commons Attribution 4.0 International license.

10.1128/mSystems.00842-20.9 TABLE S1 OGs only found in all of the euryhaline strains (see Fig. S7). 1, Fold change of the gene transcript when LTW-R cells were cultured at low salinity (only significantly affected genes are shown). 2, Mean transcript abundance (TPM) of the genes in LTW-R cells calculated from the 6 transcriptome samples (only significantly affected genes are shown). Download Table S1, DOCX file, 0.02 MB. Copyright © 2020 Xia et al. 2020 Xia et al. This content is distributed under the terms of the Creative Commons Attribution 4.0 International license.

### Transcriptomic response of LTW-R to changes in salinity.

**FIG 8:**
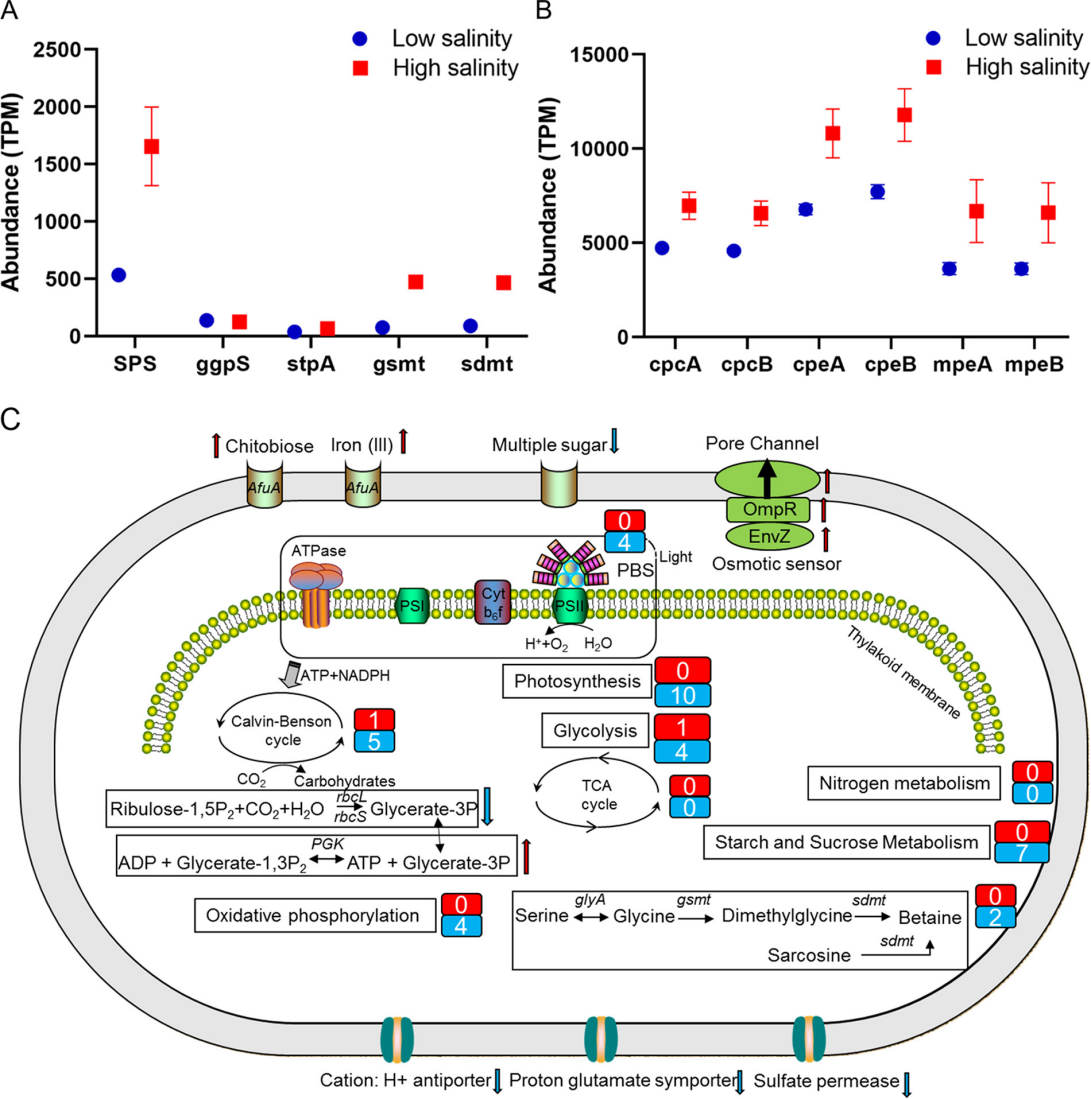
Changes in the transcript abundance of genes in LTW-R cells. (A) Genes involved in the biosynthesis of osmolytes. (B) Genes involved in the biosynthesis of key pigment proteins. (C) Overview of the response of LTW-R cells to a decrease in salinity. The numbers in the red and blue rectangles indicate the numbers of genes that were strongly upregulated and strongly downregulated, respectively. The red and blue arrows in panel C indicate the gene/pathway that was upregulated or downregulated, respectively, when salinity decreased. Genes with an abundance lower than 100 TPM in all samples were not included in panel C.

Under the low-salinity conditions, more genes involved in energy production and conversion were significantly downregulated, whereas those related to coenzyme transport and metabolism, replication, recombination, and repair, as well as amino acid transport and metabolism, were all upregulated (Fig. S8). In addition, transcripts for pigment genes, such as *mpeBA*, *cpeBA*, and *cpcBA*, decreased under the low-salinity condition (Fig. 8). We also investigated which of the LTW-R gene transcripts were strongly (fold change >2) and significantly (*P* < 0.05) affected by the decrease in salinity (Fig. 6 and 8). The results showed that 10 genes involved in photosynthesis were strongly downregulated under the low-salinity condition, whereas no genes in photosynthesis were strongly upregulated. A similar pattern was observed for the Calvin-Benson (CB) cycle as well as for starch and sucrose metabolism and oxidative phosphorylation. Our findings confirm a previous report which indicated that at lower salinity, *Synechococcus* sp. 7002 had lower transcript levels for genes encoding enzymes of the CB cycle, the inducible CO_2_-concentrating mechanism (CCM), and bicarbonate transporters (28), and are also consistent with a previous study which demonstrated that cyanobacteria *Anabaenopsis* and *Anabaena* have lower maximum photosynthetic rates in water with lower salinity (52). These observations indicate that photosynthesis and carbon metabolism in *Synechococcus* might both be negatively affected by a decrease in salinity due to a lower requirement of carbon-rich osmolytes as pathways related to the biosynthesis of osmotic components were downregulated in the lower salinity (Fig. 8A). In contrast, the *pgk* gene, which encodes phosphoglycerate kinase (PGK), was strongly increased under the low-salinity condition (Fig. 8). PGK is an ATP-generating enzyme involved in glycolysis; it catalyzes the reversible transfer of a phosphate group from 1,3-bisphosphoglycerate to ADP, producing 3-phosphoglycerate and ATP. Increasing PGK might provide more ATP for transporters to pump out the osmotic components or ions in order to maintain the osmotic balance in a low-salinity environment. An increase in the number of *pgk* gene transcripts at low salinity has also been observed in reed and cucumber seedlings (53, 54). We also found that HSP20 was strongly affected by the decrease in salinity, such that its transcripts increased 2.11-fold in LTW-R cells grown under the low-salinity conditions. Hsp20 proteins are the most abundant heat shock proteins found in plants. They function as molecular chaperones and play a vital role in plant immunity by inhibiting apoptosis and promoting both the formation of the cytoskeleton and the photosystem II (PSII) electron transport chain (55). However, the exact role of Hsp20 protein in the low-salinity adaptation of *Synechococcus* needs further study.

10.1128/mSystems.00842-20.8 FIG S8 Distribution of significantly down- or upregulated genes in the low-salinity treatments, which are grouped into different COG categories. Download FIG S8, TIF file, 2.9 MB. Copyright © 2020 Xia et al. 2020 Xia et al. This content is distributed under the terms of the Creative Commons Attribution 4.0 International license.

### Conclusions.

This study provides new insights into the mechanisms used by euryhaline PEB-containing *Synechococcus* to adapt to estuarine environments. We reveal that S5.2 *Synechococcus* have developed different pigment types while retaining their ability to deal with salinity changes, which highly expands their niche in estuarine environments. In addition, we are the first to report a high-quality genome of the novel S5.1 *Synechococcus* clade HK1. Strains of this clade were very effective at dealing with salinity changes, indicating that some lineages of S5.1 *Synechococcus* might also adapt well to riverine affected waters. Using a combination of genomic and transcriptomic analysis, we identified mechanisms used by euryhaline *Synechococcus* for adapting to estuarine environments, and we found the loss of some genes might explain why strictly marine *Synechococcus* are unable to grow at low salinity (i.e., salinity of <15 ppt). This may be because the *glzT* gene (named by this study) encodes a transmembrane protein that is present only in the genome of euryhaline strains, and this plays a key role in their ability to adapt to low salinity. On the other hand, to adapt to high-salinity conditions, euryhaline strains often use sucrose as an osmolyte, whereas oceanic strains prefer betaine. However, the euryhaline LTW-R is more similar to S5.1 oceanic *Synechococcus* strains than it is to the S5.2 euryhaline PCB-only strain in that it has *sdmt* and *gsmt* genes and uses betaine and sucrose as its osmotic components. LTW-R also contains the pigment protein PUB, and so together these characteristics might help it expand to less turbid coastal waters. Finally, although the transcript levels of photosynthesis-related genes were significantly decreased under the low-salinity condition, LTW-R still maintained a relatively high rate of growth. This is likely to be possible due to a decrease in the requirement for the biosynthesis of osmotic components. These results explained why *Cyanobacteria* (e.g., *Anabaenopsis* and *Anabaena*) have lower maximum photosynthetic rates in water with lower salinity. We also observed that several highly expressed genes were strongly upregulated in the low-salinity treatments; however, their function remains unknown. In future studies, a combination of cell sorting and next-generation DNA sequencing techniques would be useful for exploring the diversity of euryhaline PEB-containing *Synechococcus* in estuarine environments in more detail.

## MATERIALS AND METHODS

### Determining the distribution of PEB-containing and PCB-only *Synechococcus* in the Pearl River estuary.

Samples for counting PEB-containing and PCB-only *Synechococcus* abundance were collected from the Pearl River estuary on a cruise conducted from 13 to 20 July 2014 (Fig. 1). Samples (1.8 ml) of water from each station were fixed with seawater-buffered paraformaldehyde (0.5%, final concentration), flash frozen in liquid nitrogen, and stored at −80°C. PEB-containing *Synechococcus* cells were enumerated using a Becton Dickinson FACSCalibur flow cytometer equipped with dual lasers (488 and 635 nm) with a high flow rate, following the method described by Liu et al. (56). Ten microliters of yellow-green fluorescent beads (1-μm diameter; Polysciences, Warrington, PA, USA) was added to each sample as an internal standard. Flow cytometric data were analyzed using WinMDI software 2.9 (Joseph Trotter, Scripps Research Institute, La Jolla, CA, USA).

### Isolation of *Synechococcus* strains.

*Synechococcus* sp. strains HK01 and LTW-R were isolated from PM7 (114.295°E, 22.342°N) and LTW (114.129°E, 22.223°N) stations, respectively. Annually, the salinity of the surface waters at PM7 and LTW ranged from 18.2 to 33.8 ppt and from 19.3 to 34.0 ppt, respectively. Water samples were filtered through a 1-μm polycarbonate (PC) membrane (Pall Corporation, New York, USA), and then each 1-ml sample of filtered water was added to 3 ml modified f/2 medium (57) (without Na_2_SiO_3_·9H_2_O but containing 100 μM NH_4_Cl), which was diluted 5 times with seawater. The water samples were incubated at 25°C under illumination of ∼20 μmol quanta m^−2^ s^−1^ in a 12-h/12-h light-dark cycle for 30 days until the cultures were slightly pink or green. To obtain monoclonal cultures, we performed seven 10-fold serial dilutions with 1-ml aliquots of each culture. These diluted cultures were then incubated under the conditions described above, for a further 2 months. After two additional rounds of purification by the same serial dilution method, *Synechococcus* strains were identified by amplification of the *rpoC1* gene (10). Cultures that had different *rpoC1* sequences were purified further by the serial dilution method.

### Growth of *Synechococcus* isolates under different salinities.

For salinity shock experiments, six *Synechococcus* strains including HK01, LTW-R, CC9605, WH8102, WH7803, and WH5701 were grown in the modified f/2 medium for 8 days (in the exponential phase), and then they were transferred to fresh modified f/2 medium with 4 different salinities (14 ppt, 24 ppt, 34 ppt, and 44 ppt; prepared using different NaCl concentrations). The cultures were then incubated under the conditions described above, and the absorbance at 440 nm was measured every day for 22 consecutive days (10), in order to determine the growth rate of the *Synechococcus* cultures.

### *In vivo* absorption spectra of HK01 and LTW-R.

The *in vivo* absorption spectra of the *Synechococcus* cultures were measured as described previously (10). In brief, an aliquot of the exponentially growing culture was transferred to a cuvette, and the *in vivo* absorption spectrum was measured from 400 to 700 nm using a spectrophotometer (UH5300; Hitachi, USA), with a scan rate of 2 nm s^−1^. The spectra were normalized at 440 nm.

### DNA extraction, genome sequencing, and assembly.

For DNA extraction, *Synechococcus* strains were incubated in the modified f/2 medium for 10 days (in the exponential phase), and then the cells were filtered onto 0.2-μm PC membranes. The membranes were cut into 3-mm- by 3-mm-size squares, and then DNA was extracted using a DNA extraction minikit (Invitrogen, Thermo Fisher Scientific, Carlsbad, CA, USA) following the manufacturer’s instructions. Genomic DNA was sequenced using an Illumina HiSeq 2000 sequencing system (Shanghai South Gene Company, Shanghai, China) and a PacBio system (Guangzhou Magigene Company, Guangzhou, China). Details about the sequence information are listed in Data Set S1 in the supplemental material. The cyanobacterial genomes were assembled from a combination of the Illumina HiSeq 2000 and PacBio clean reads using Unicycler with the default setting (58). Contigs that were longer than 2 kb were identified using the NR database, and those affiliated with *Cyanobacteria* were retained for subsequent analysis. The LTW-R genome comprises a single circular chromosome, and HK01 has a single contig.

### Annotation, subsystem analysis, and phylogeny analysis.

For annotation, the genome sequences of HK01 and LTW-R were submitted to the RAST server for open reading frame (ORF) prediction (59). Predicted ORFs and amino acid sequences were annotated using the eggNOG-Mapper v2 with default settings (60) and BlastKOALA (61). KEGGMAPPER (http://www.genome.jp/kegg/mapper.html) was then used to reconstruct the metabolic pathways. The two-component systems and ABC transporters in the HK01 and LTW-R genomes were identified by BlastKOALA as this is more sensitive than eggNOG-Mapper. For the phylogenetic analysis of *Synechococcus* strains, 44 high-quality genome sequences were downloaded from the NCBI database. A maximum likelihood (ML) phylogenetic tree of *Synechococcus* was then constructed based on 43 concatenated phylogenomic markers of the *Synechococcus* isolates (for details of the phylogenetic markers, please see Table S6 of reference 32) and reference genomes using the CheckM (32) and MEGA6 (62) software packages.

### Prediction of gene gain or loss events among the *Synechococcus* genomes.

To predict gene gain or loss events among the *Synechococcus* genomes, ORFs of LTW-R and HK01, as well as the 44 reference genomes, were predicted using the RAST server, after which orthologues of the *Synechococcus* genomes were identified using OrthoFinder (63). Gene gain or loss events were further predicted using the Count software package (64). For each gene family, Wagner parsimony with gene gain penalty of 1 was used to infer the most parsimonious ancestral gene sets with different gain/loss pressures. Orthologous clusters of six complete *Synechococcus* genomes (HK01 [HK1], CC9605 [clade II], WH8102 [clade III], WH7803 [clade V], LTW-R [S5.2], and CB0101 [S5.2]) were identified using OrthoVenn with a cutoff E value of 1e−5 (65). To determine horizontal gene transfer (HGT) of a pigment gene among the *Synechococcus* strains, we constructed a species tree and a gene tree based on the genome sequences and *cpeB* gene (encoding phycoerythrobilin protein) sequences, respectively. HGT detection was then used to infer and validate horizontal gene transfer events (66).

### Membrane spans prediction and *Synechococcus* pigment operon comparison.

For membrane spans prediction, amino acid sequences of LTW-R were obtained and submitted to TMpred (https://embnet.vital-it.ch/software/TMPRED_form.html), an algorithm designed to predict transmembrane helices from protein sequences. To obtain the pigment operon of HK01 and LTW-R, annotated genomes were imported into Geneious V9 (Biomatters, Auckland, New Zealand), and the pigment operon sequences were extracted and then compared with reference *Synechococcus* strains using ChromoMapper (https://www2.unil.ch/biomapper/chromomapper/).

### Transcriptomic analysis of *Synechococcus* sp. LTW-R under different salinities.

LTW-R was grown in the modified f/2 medium for 8 days (exponential phase) and then transferred to fresh medium with a salinity of either 10 ppt (treatment) or 33 ppt (control). After acclimation for 8 days, the treatment and control groups were transferred to fresh medium with a salinity of 10 ppt or 33 ppt, respectively. After incubation for a further 8 days, the cells were collected using 0.2-μm PC membranes and immersed in RNAlater (Ambion). Both treatment and control were prepared and incubated in triplicate in 1-liter Nalgene bottles. RNA was extracted using TRIzol (Invitrogen, China) according to the manufacturer’s instructions and then sent to Guangzhou Magigene Company (Guangzhou, China) for library construction and sequencing. For transcriptomic analysis, low-quality sequences were removed using Trimmomatic (67), and then sequence assembly, gene prediction, and annotation were conducted using the SqueezeMeta pipeline in the default setting (68). In addition to the COG and KEGG databases, a database constructed from the annotated amino acid sequences of LTW-R was used to annotate the transcriptome sequences. The expression values of each LTW-R gene were calculated from the uniquely mapped reads using the “transcript per million” (TPM) approach (69). Expression levels of each gene were compared using DESeq2 (70). Differences between the corresponding controls and treatments were considered to be statistically significant at *P* < 0.05. Transcripts with a fold change of >2 were considered to be strongly up- or downregulated.

### Sequencing information and accession numbers.

Information regarding the Illumina HiSeq 2000 and PacBio sequencing data is shown in Data Set S1. The HK01 and LTW-R genomes were submitted to the NCBI database with accession numbers CP059059 and CP059060, respectively. In addition, the Illumina HiSeq 2000 and PacBio sequences were submitted to the NCBI Sequence Read Archive with BioProject accession number PRJNA645008.
