# Supplementary figures and images for: Discovery of Euryhaline Phycoerythrobilin-Containing Synechococcus and Its Mechanisms for Adaptation to Estuarine Environments
Source: mSystems. 2020 Dec 15;5(6):e00842-20. doi: 10.1128/mSystems.00842-20 (PMC7771541; doi:10.1128/mSystems.00842-20)

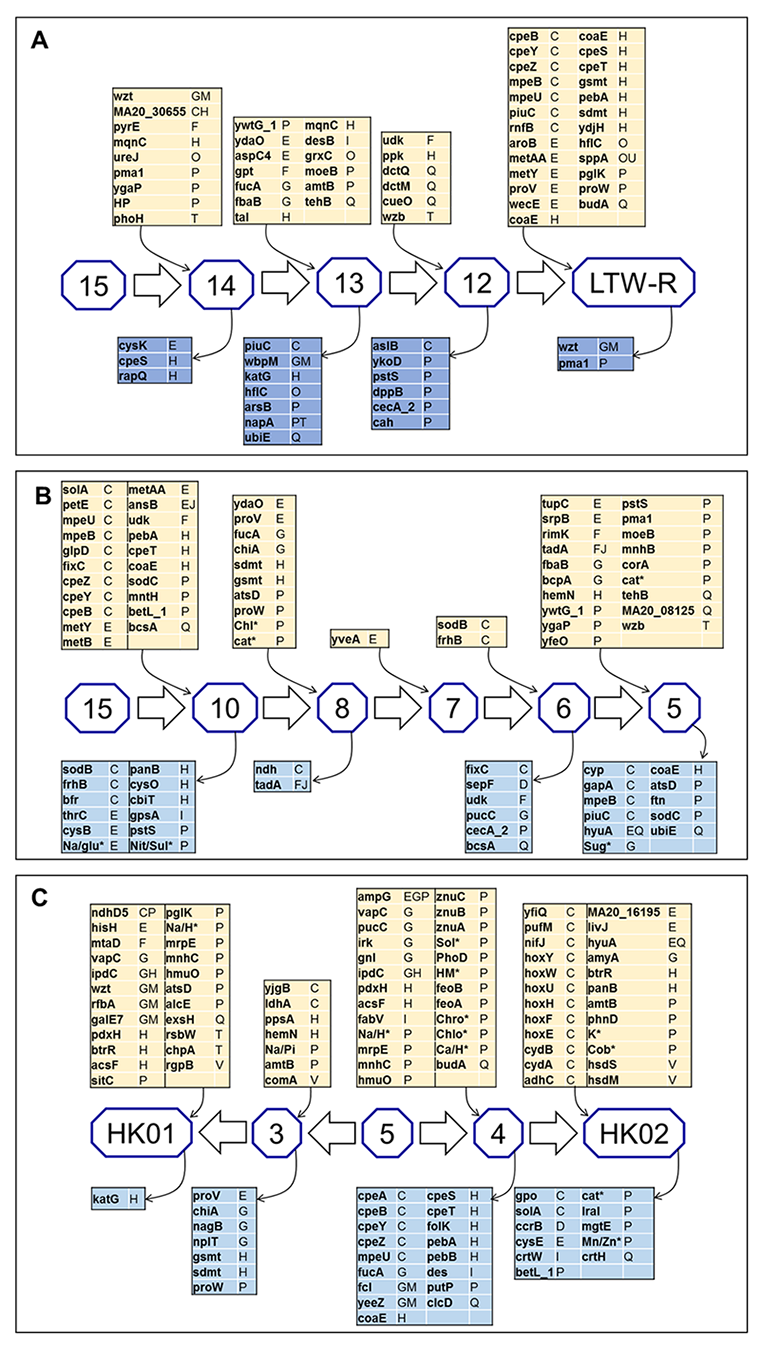

Supplement: FIG S1 [file mSystems.00842-20-sf001.tif]

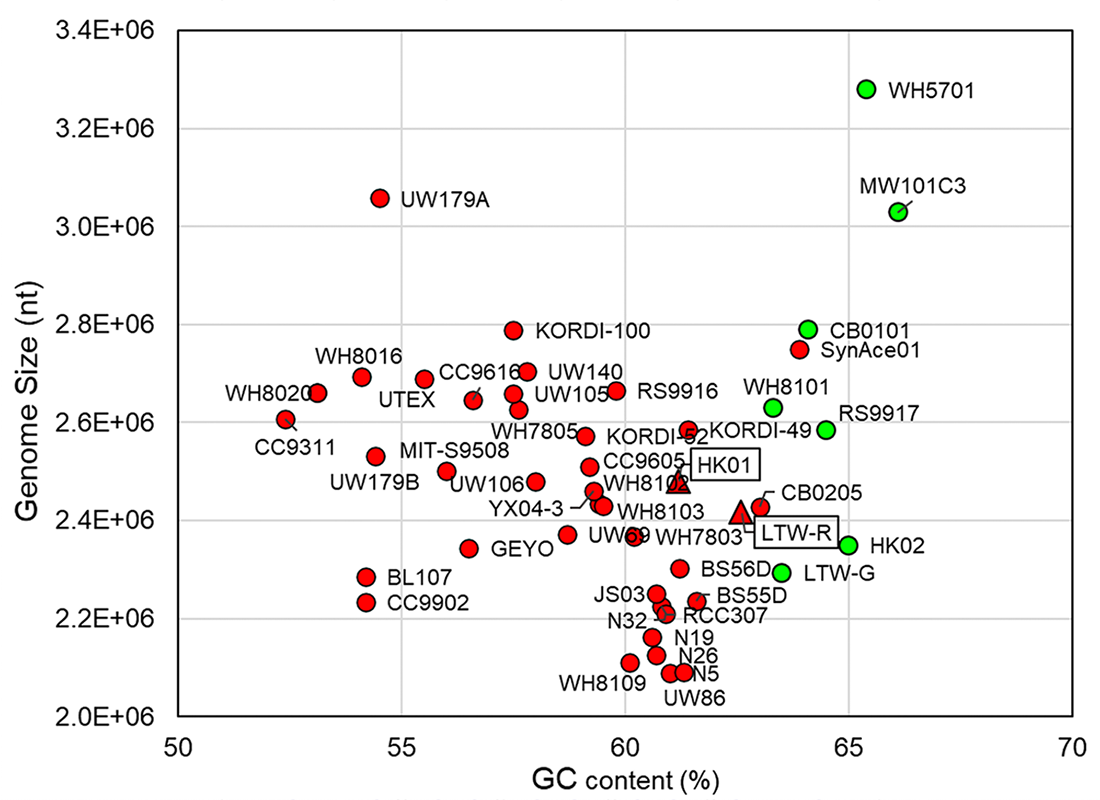

Supplement: FIG S2 [file mSystems.00842-20-sf002.tif]

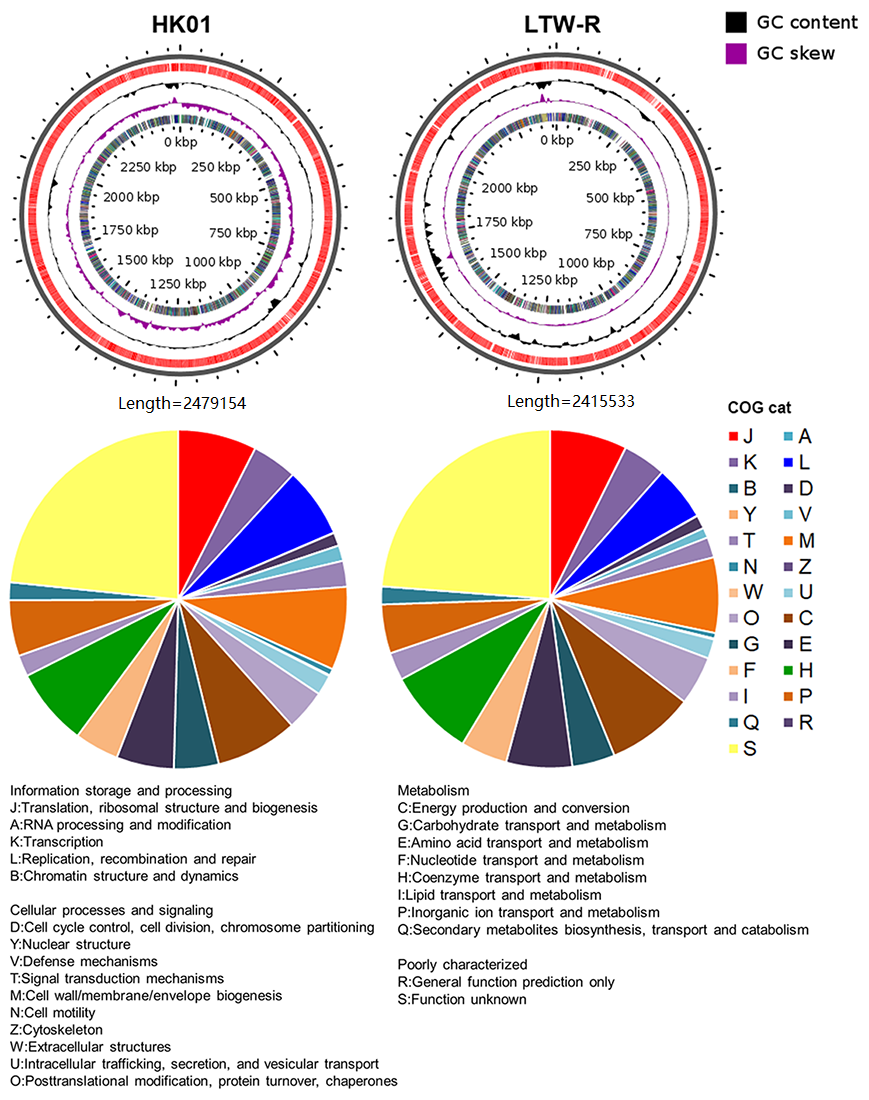

Supplement: FIG S3 [file mSystems.00842-20-sf003.tif]

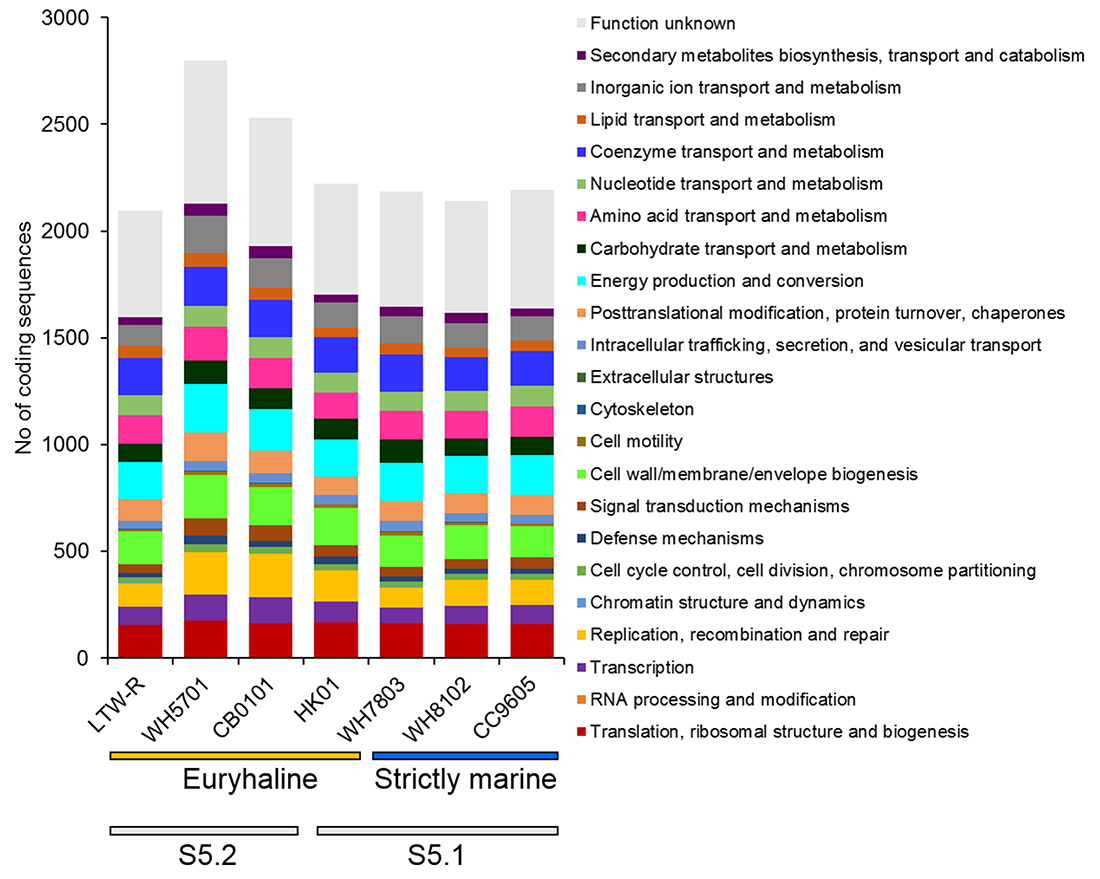

Supplement: FIG S4 [file mSystems.00842-20-sf004.tif]

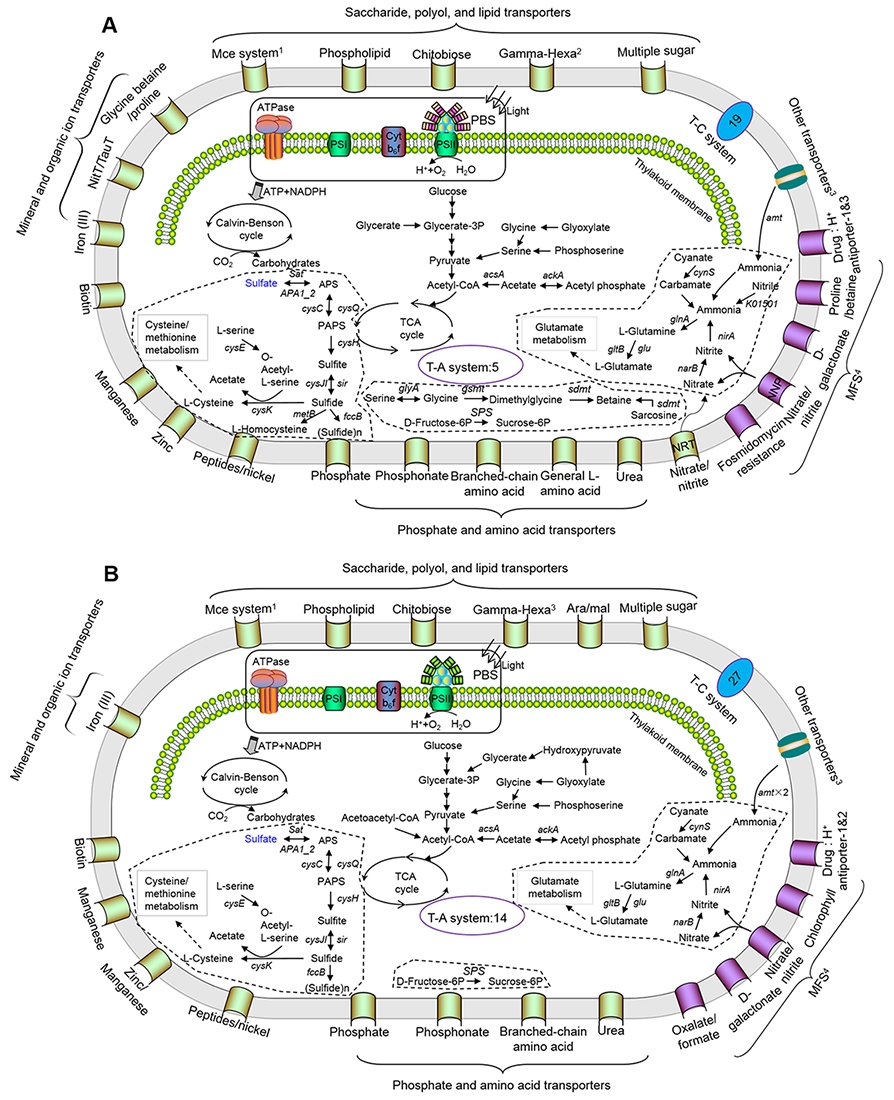

Supplement: FIG S5 [file mSystems.00842-20-sf005.tif]

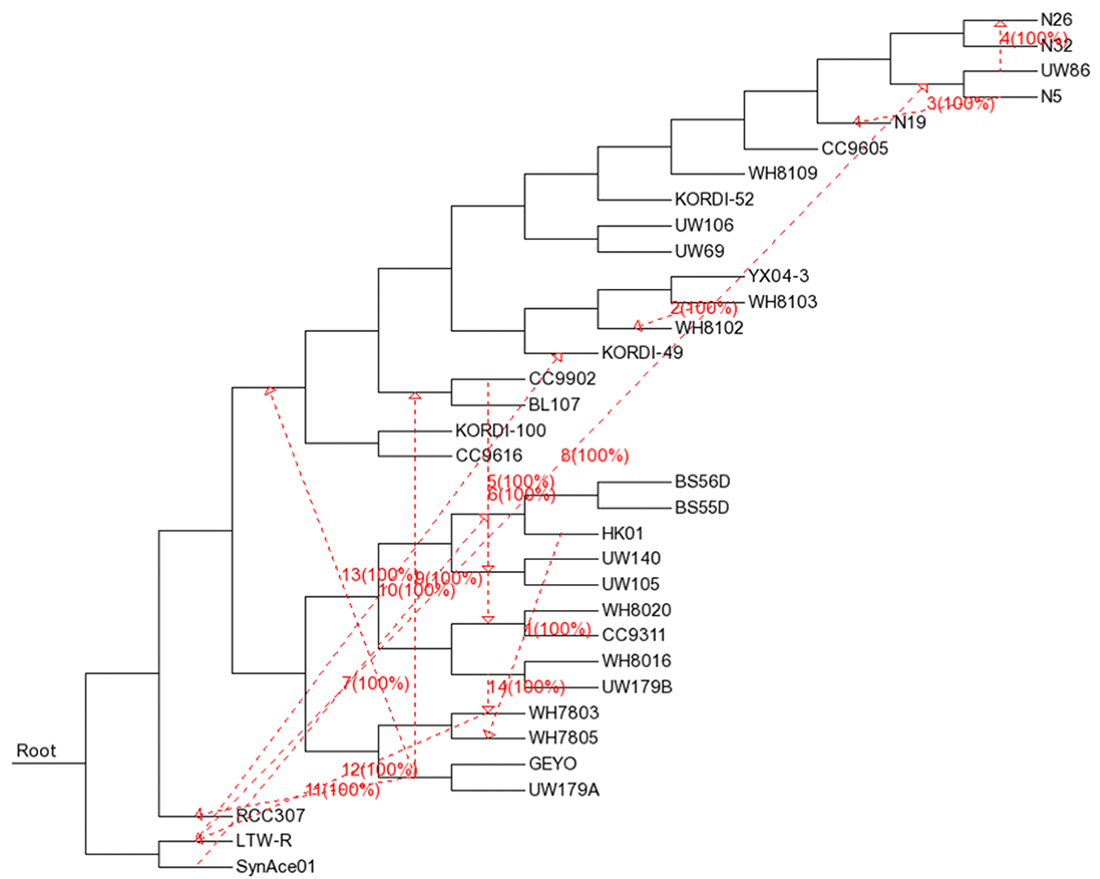

Supplement: FIG S6 [file mSystems.00842-20-sf006.tif]

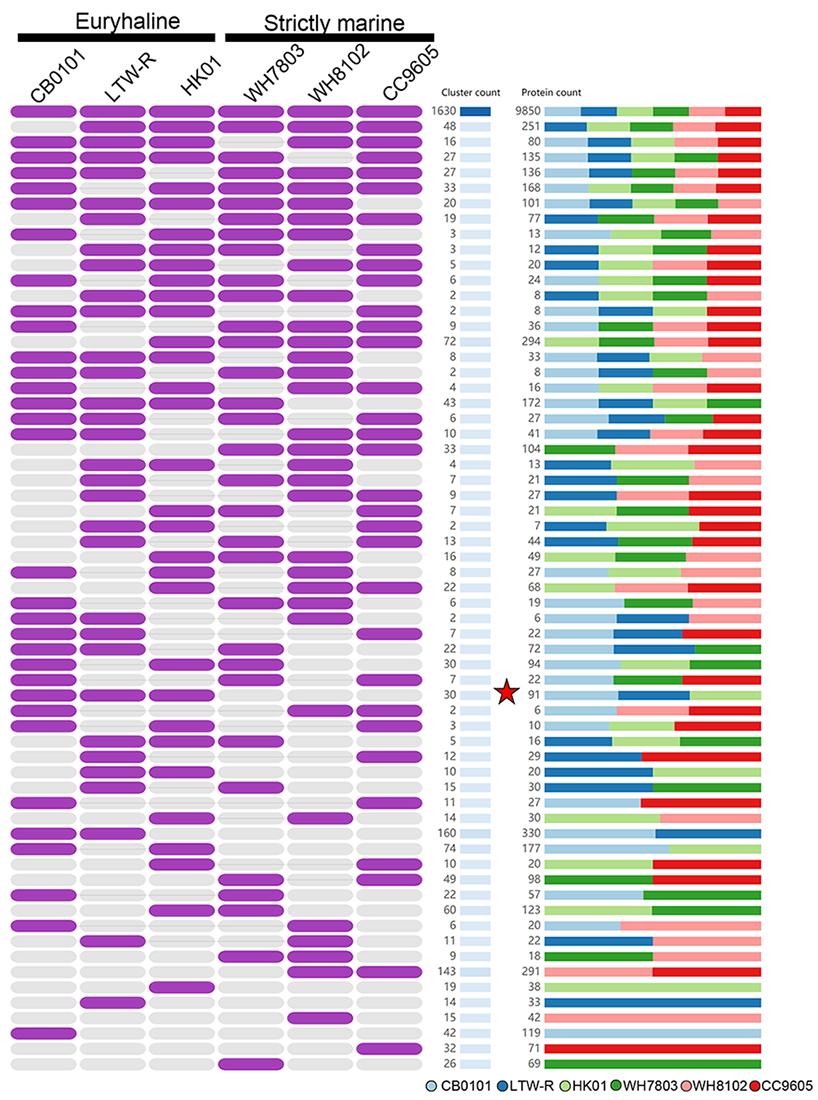

Supplement: FIG S7 [file mSystems.00842-20-sf007.tif]

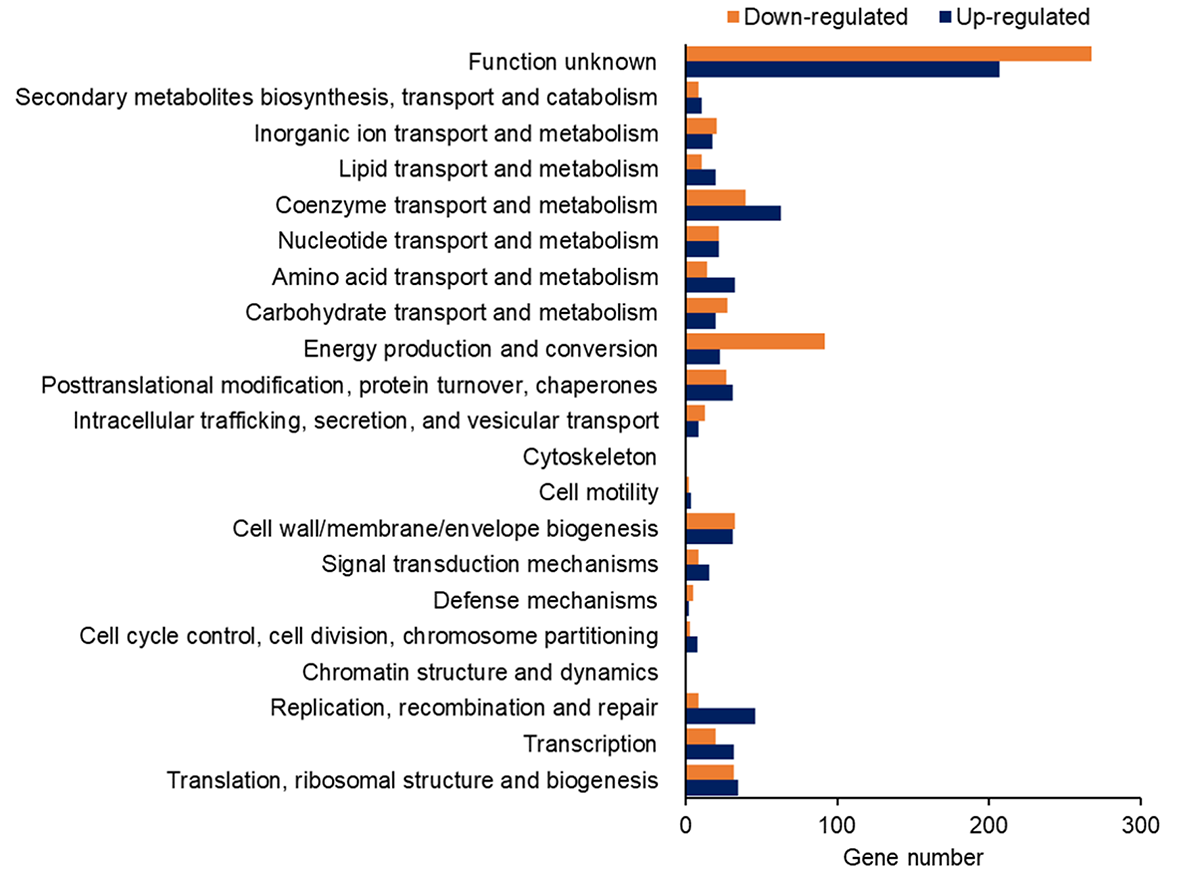

Supplement: FIG S8 [file mSystems.00842-20-sf008.tif]
