# Supplementary material for: Discovery of Euryhaline Phycoerythrobilin-Containing Synechococcus and Its Mechanisms for Adaptation to Estuarine Environments
Source: mSystems. 2020 Dec 15;5(6):e00842-20. doi: 10.1128/mSystems.00842-20 (PMC7771541; doi:10.1128/mSystems.00842-20)
Supplement: TABLE S1 [file mSystems.00842-20-st001.docx]

Table S1. OGs only found in all of the euryhaline strains (see Figure S7). ^1^, Fold-change of the gene transcript when LTW-R cells were cultured at low salinity (only significantly affected genes are shown). ^2^, Mean transcript abundance (TPM) of the genes in LTW-R cells calculated from the 6 transcriptome samples (only significantly affected genes are shown).

| OG_name | Go_annotation | Ko number | Gene_name | Function | LTW-R ORF | Fold change^1^ | Mean abundance (TPM)^2^ |
| --- | --- | --- | --- | --- | --- | --- | --- |
| cluster1840 | GO:0009617; P:response to bacterium; HEP:dictyBase | K01915 | glnA, GLUL | glutamine synthetase | LTW-RLTW-R.991 |  |  |
| cluster2161 | N/A | N/A | N/A | N/A | LTW-RLTW-R.358 |  |  |
| cluster2186 | N/A | N/A | N/A | N/A | LTW-RLTW-R.1416 |  |  |
| cluster2188 | GO:0016829; F:lyase activity; IEA:UniProtKB-KW | K02289 | cpcF | phycocyanobilin lyase subunit beta | LTW-RLTW-R.1636 |  |  |
| cluster2191 | N/A | K03192 | ureJ | urease accessory protein | LTW-RLTW-R.44 |  |  |
| cluster2193 | GO:0015341; F:zinc efflux active transmembrane transporter activity; IBA:GO_Central | K16264 | czcD, zitB | cobalt-zinc-cadmium efflux system protein | LTW-RLTW-R.1158 |  |  |
| cluster2194 | GO:0035435; P:phosphate ion transmembrane transport; ISO:PomBase | K08176 | PHO84 | MFS transporter, PHS family, inorganic phosphate transporter | LTW-RLTW-R.1025 |  |  |
| cluster2196 | N/A | N/A | N/A | glycine zipper 2TM domain-containing protein | LTW-RLTW-R.1182 | 9.06 | 3634.02 |
| cluster2197 | N/A | N/A | N/A | N/A | LTW-RLTW-R.422 |  |  |
| cluster2202 | N/A | K08680 | N/A | N/A | LTW-RLTW-R.388 | 2.18 | 58.53594 |
| cluster2204 | N/A | K03892 | arsR | ArsR family transcriptional regulator, arsenate/arsenite/antimonite-responsive transcriptional repressor | LTW-RLTW-R.241 |  |  |
| cluster2210 | N/A | K16868 | tehB | tellurite methyltransferase | LTW-RLTW-R.240 |  |  |
| cluster2212 | GO:0008757; F:S-adenosylmethionine-dependent methyltransferase activity; IEA:UniProtKB-UniRule | K17462 | yrrT | putative AdoMet-dependent methyltransferase | LTW-RLTW-R.1468 |  |  |
| cluster2213 | GO:0001522; P:pseudouridine synthesis; IEA:InterPro | K06181 | rluE | 23S rRNA pseudouridine2457 synthase | LTW-RLTW-R.2553 | 2.03 | 217.6084 |
| cluster2214 | N/A | N/A | N/A | N/A | LTW-RLTW-R.760 |  |  |
| cluster2216 | GO:0005975; P:carbohydrate metabolic process; IEA:InterPro | N/A | xfp | N/A | LTW-RLTW-R.1999 |  |  |
| cluster2218 | GO:0009243; P:O antigen biosynthetic process; IEA:UniProtKB-UniPathway | N/A | N/A | N/A | LTW-RLTW-R.1841 |  |  |
| cluster2222 | N/A | N/A | N/A | N/A | LTW-RLTW-R.213 |  |  |
| cluster2225 | GO:0070221; P:sulfide oxidation, using sulfide:quinone oxidoreductase; TAS:Reactome | K17725 | moeB, ETHE1 | sulfur dioxygenase | LTW-RLTW-R.239 |  |  |
| cluster2231 | N/A | K07090 | K07090 | uncharacterized protein | LTW-RLTW-R.237 | 3.37 | 167.2823 |
| cluster2232 | N/A | N/A | N/A | N/A | LTW-RLTW-R.252 |  |  |
| cluster2237 | N/A | K07267 | oprB | porin | LTW-RLTW-R.1033 |  |  |
| cluster2245 | N/A | N/A | N/A | N/A | LTW-RLTW-R.1128 |  |  |
| cluster2246 | GO:0005886; C:plasma membrane; IEA:UniProtKB-SubCell | K03321 | ygaP, TC.SULP | sulfate permease, SulP family | LTW-RLTW-R.238 |  |  |
| cluster2249 | GO:0016829; F:lyase activity; IEA:UniProtKB-KW | K02288,K02631 | cpcE | N/A | LTW-RLTW-R.1637 |  |  |
| cluster2250 | N/A | N/A | N/A | N/A | LTW-RLTW-R.1043 |  |  |
| cluster2251 | N/A | N/A | N/A | N/A | LTW-RLTW-R.368 |  |  |
| cluster2257 | N/A | N/A | N/A | N/A | LTW-RLTW-R.211 |  |  |
| cluster2258 | N/A | N/A | N/A | N/A | LTW-RLTW-R.617 |  |  |
| cluster2265 | GO:0008519; F:ammonium transmembrane transporter activity; IEA:InterPro | K03320 | amt, AMT, MEP | ammonium transporter, Amt family | LTW-RLTW-R.2503 |  |  |
